# Melanoma Plasticity: Promoter of Metastasis and Resistance to Therapy

**DOI:** 10.3389/fonc.2021.756001

**Published:** 2021-09-16

**Authors:** Fan Huang, François Santinon, Raúl Ernesto Flores González, Sonia V. del Rincón

**Affiliations:** ^1^Lady Davis Institute, McGill University, Montréal, QC, Canada; ^2^Department of Experimental Medicine, McGill University, Montréal, QC, Canada; ^3^Department of Oncology, McGill University, Montréal, QC, Canada

**Keywords:** melanoma, phenotype switching, targeted therapy, immunotherapy, therapy resistance

## Abstract

Melanoma is the deadliest form of skin cancer. Although targeted therapies and immunotherapies have revolutionized the treatment of metastatic melanoma, most patients are not cured. Therapy resistance remains a significant clinical challenge. Melanoma comprises phenotypically distinct subpopulations of cells, exhibiting distinct gene signatures leading to tumor heterogeneity and favoring therapeutic resistance. Cellular plasticity in melanoma is referred to as phenotype switching. Regardless of their genomic classification, melanomas switch from a proliferative and differentiated phenotype to an invasive, dedifferentiated and often therapy-resistant state. In this review we discuss potential mechanisms underpinning melanoma phenotype switching, how this cellular plasticity contributes to resistance to both targeted therapies and immunotherapies. Finally, we highlight novel strategies to target plasticity and their potential clinical impact in melanoma.

## Introduction

**Figure 1:**
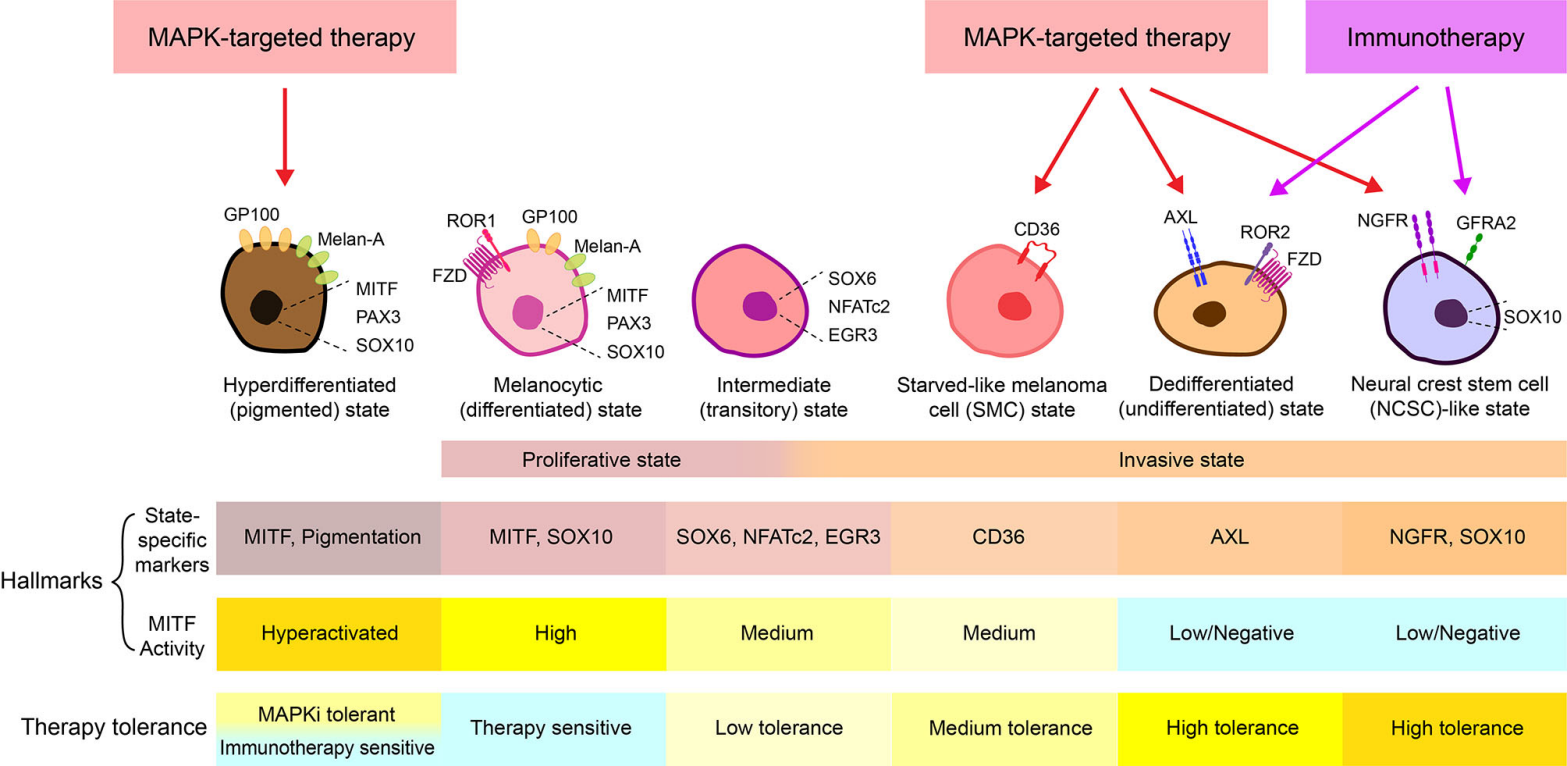
Melanoma cell states. At least 6 different melanoma cell states have been thus far characterized, including a MAPKi-induced hyperdifferentiated/pigmented state, a MITF^high^/AXL^low^ melanocytic/differentiated state, an intermediate/transitory state, a CD36^+^ starved-like melanoma cell (SMC) state, a MITF^low^/AXL^high^ dedifferentiated/undifferentiated state, and a MITF^low^/NGFR^high^ neural crest stem cell (NCSC)-like state (3, 13–16). While the hyperdifferentiated state is induced by MAPK-targeted therapy and the intermediate state exhibit both proliferative and invasive phenotypes, the melanocytic state generally corresponds to the “proliferative” state, and the SMC state, the dedifferentiated state, and the NCSC-like state together make up the “invasive” state (14–16). Notably, in many cases, an “invasive” phenotype is used to describe the MITF^low^/AXL^high^ dedifferentiated population, while in some other cases, the “invasive” state refers to both AXL^high^/dedifferentiated and NGFR^high^/dedifferentiated populations. To avoid confusion, cell state-specific markers and MITF activity are often combined to define each state. For example, it is generally accepted that the melanocyte state is marked as MITF^high^/AXL^low^, the SMC state is marked with CD36^+^ and medium activity of MITF, the dedifferentiated state is defined as MITF^low^/AXL^high^ and the NCSC-like state is defined as MITF^low^/NGFR^high^/SOX10^+^/GFRA2^+^ (3, 13–16).

Melanoma is the deadliest form of skin cancer due to its high metastatic potential. Although MAP-Kinase pathway (MAPK)-targeted therapies and immunotherapies have revolutionized the management of patients with metastatic melanoma, their clinical benefit is limited by the almost inevitable development of resistance and tumor recurrence. Metastasis and therapy resistance of numerous tumor types is associated with intratumoral heterogeneity and cancer cell plasticity (1–5). Melanoma has been well described to comprise phenotypically distinct subpopulations of cells. Gene expression analyses of cultured melanoma cells identified two predominant cell populations, exhibiting either ‘proliferative’ or ‘invasive’ phenotypes (6–9), reminiscent of the intratumoral heterogeneity present in patient-derived melanomas (10, 11). Throughout this review we will refer to a two-state system, the proliferative state/phenotype which is described as “differentiated”, “epithelial-like”, with high expression of microphthalmia-associated transcription factor (MITF) as a hallmark (MITF^high^/AXL^low^), while the invasive phenotype is described as “undifferentiated/dedifferentiated”, “mesenchymal-like”, with a marked expression of the receptor tyrosine kinase AXL (MITF^low^/AXL^high^) (10, 12, 13). Over the years, additional cell states have been defined with unique gene expression signatures, and differential therapeutic sensitivity and metastatic potential associated with each of these phenotypes (**Figure 1**) (14–16).

Similar mutations and gene expression patterns present in primary and metastatic melanomas suggest a mechanism independent of clonal evolution as a major driver of melanoma progression (17–21). An alternative concept of cancer stem cells provides one explanation for the phenotypic and functional heterogeneity among cancer cells in some tumors (2, 22). This model describes a hierarchy of intratumoral subpopulations comprising mainly tumor cells that do not form tumors when implanted into immunodeficient mice and a rare population of cancer cells with stem cell-like properties, which are thought to drive tumor progression, tumor dissemination, and therapy resistance (2, 22). Studies have shown however that all melanoma cells, and not just a small subset of stem-like cells, possess tumor initiating potential and can restore phenotypic heterogeneity when injected into immunodeficient mice, suggesting non-hierarchical plasticity of melanoma cells (23, 24). Notably, in the above-mentioned experimental settings, single melanoma cells were artificially implanted into immunocompromised mice, and subsequently formed tumors with restored heterogeneity (23, 24). However, for a tumor-initiating cell to drive metastasis, it needs to be invasive. In most cases, metastases are driven by several tumor cells in a cooperative manner and heterogeneity is therefore maintained and reprogrammed at the metastatic site (13, 22). Thus, a new paradigm termed “phenotype switching” emerged to better describe the plasticity of melanoma cells and their stem-like behavior. This model predicts that melanoma metastasis and phenotypic heterogeneity is driven by specific gene expression programs rather than by the accumulation of irreversible genetic events. Microenvironmental conditions, coupled with melanoma cell-intrinsic pathways, regulate melanoma cell switching between a proliferative state or a mesenchymal-like invasive state (22). In line with this paradigm, the ‘proliferative, differentiated, and often therapy-sensitive’ and ‘invasive, dedifferentiated/undifferentiated, and often therapy-resistant’ melanoma cells can co-exist in bulk tumor tissues (10, 11) and are not defined by irreversible genetic lesions (6, 25). Rather, melanoma cells are phenotypically plastic, or ‘phenotype switch’, *in vitro* and *in vivo* (6, 15, 25, 26), *via* a process akin to the reversible epithelial-to-mesenchymal transition (EMT), which is characteristic of epithelial tumors.

Hyperactivation of tumor cell-intrinsic MAPK and PI3K signaling, microenvironmental stress conditions (i.e., hypoxia, nutrient limitation, and chronic inflammation) are commonly observed in melanomas of all genomic classifications. These varied growth conditions induce several stress adaptive pathways in melanoma cells, such as the HIF1α pathway, p38 MAPK pathway, and integrative stress response (ISR), which are generally believed to be essential drivers of phenotype switching. Frontline therapies, such as MAPK-targeted therapy and immunotherapy, can further induce these common stress signals. Together, these factors cooperate to promote melanoma phenotype switching, which will ultimately determine the invasive potential and therapeutic response regardless of their genomic mutations (14, 16). Therefore, blocking phenotypic switching is a promising and universal strategy in melanoma to prevent metastasis and overcome drug-resistance.

Here, we dissect mechanisms underpinning melanoma phenotype switching in response to a variety of stress conditions and their link to therapy resistance. We highlight recent studies demonstrating novel strategies to target plasticity and their potential clinical impact in melanoma. While there are a number of subtypes of melanoma, including acral and mucosal, in this review we focus on cutaneous melanoma, with a short discussion of recent reports of plasticity in uveal melanoma.

## Mechanisms Underpinning Melanoma Phenotype Switching

For the past 20 years, several studies on melanoma plasticity have focused on characterizing gene expression signatures and transcriptional programs of the proliferative versus the invasive states of melanoma cells (3, 13, 27). These studies have provided key insights into the molecular mechanisms driving phenotype switching and their relationship to metastasis and therapy resistance.

### Overview of Phenotype Switching Associated Gene Signatures

The melanocyte lineage is derived from the neural crest through delamination and the epithelial-to-mesenchymal transition (EMT), both critical processes for embryonic morphogenesis and lineage differentiation. The invasive and proliferative phenotype gene signatures describe at least two distinct melanoma cell states resembling different phases of melanocyte lineage development, ranging from neural-crest stem cells (NCSCs) to highly differentiated melanocytes (**Figure 1**) (14, 16).

In the classic two-state system, ‘proliferative’ melanoma cells are thought to reflect those that proliferate rapidly in optimal low-stress conditions, such as within a suitable metastatic niche (28, 29). They exhibit a clear differentiation phenotype, marked with high expression of MITF, a master regulator of melanocyte lineage differentiation and hallmark of the proliferative melanoma cell phenotype (12). In line with the role of MITF in regulating the proliferative state, its upstream regulators (*SOX10*, *PAX3*, *EDNRB* and *CREB*) are often regarded as drivers of the invasive-to-proliferative switch, and its downstream targets (*MLANA*, *PMEL*, *DCT, TYRP*) are known markers of the proliferative signature (27, 30).

While MITF^low^-marked undifferentiation/dedifferentiation is generally accepted as a key feature of the invasive phenotype, the “invasive” gene signature appears to be more complex. Diverse signaling pathways that allow melanoma cells to adapt to a variety of high-stress conditions, have been associated with the invasive state. In line with the theory that phenotype switching is largely driven through microenvironmental-induced transcriptional changes (3, 7, 8, 13, 28, 31), the proposed “invasive” signature includes multiple extracellular factors, membrane receptors, transcriptional regulators, epigenetic factors, and their downstream targets and/or effectors, which are summarized in **Table 1**. Interestingly, proteins of the same family are sometimes found inversely expressed in melanoma cells with distinct phenotypes. For example, the switch in expression between pairs of closely related transcriptional regulators, such as ZEB2/ZEB1 (35–38), SOX10/SOX9 (32, 33), and LEF1/TCF4 (34), drive melanoma cells towards the proliferative and invasive phenotype, respectively.

**Table 1:** Melanoma cell state-specific gene signature.

Signature	Type	Protein/Gene name & description
Proliferative Signature	Transcription factors (TFs)	MITF/*MITF*: Hallmark of the proliferative melanoma signature (12, 14, 16) SOX10/*SOX10*: Upstream TF of *MITF (*14, 16, 27, 30, 32, 33) PAX3/*PAX3*: Upstream TF of *MITF (*27, 30) CREB/*CREB1*: Upstream TF of *MITF (*27, 30) LEF1/*LEF1*: β-catenin co-factor, suppresses TCF4 expression (34) ZEB2/*ZEB2*: The ZEB1-to-ZEB2 switch promotes the proliferative switch (35–38)
	Receptors	EDNRB/*EDNRB*: Upstream regulator of MITF, Endothelin-3 receptor (27, 30) ROR1: The ROR2-to-ROR1 switch promotes the proliferative switch (39)
	Extracellular ligands	Endothelin-3/*EDN3*: Upstream regulator of MITF; EDNRB ligand (28, 40)
	MITF targets	Melan-A/*MLANA*: Melanocytic antigen GP100/*PMEL*: Melanocytic antigen Others: Tyrosinase/*TYR*, TYRP2/*DCT*, TYRP1/*TYRP1*
Invasive Signature	Transcription factors	BRN2/*POU3F2*: Pan-signal-induced regulator of phenotype switching (41–47) c-Jun/*JUN*: Activator Protein-1 (AP-1) transcription factor subunit (48–50) JunB/*JUNB*: AP-1 transcription factor subunit (51, 52) Fra1/*FOSL1*: AP-1 transcription factor subunit (53) TEADs/*TEAD1*, *TEAD2*, *TEAD3*, *TEAD4 (*25, 54) HIF1α/*HIF1A*: Key regulator of hypoxia-induced phenotype switching (8, 39, 55, 56) ATF4/*ATF4*: Key regulator of starvation-induced phenotype switching (9, 57) NF-κB/*NFKB1*: Key regulator of inflammation-induced phenotype switching (48, 58–60) HOXA/*HOXA* (61) ZEB1/*ZEB1*: The ZEB2-to-ZEB1 switch promotes the invasive switch (35–38) SOX9/*SOX9* (33) TCF4/*TCF4*: β-catenin co-factor, inversely correlated with LEF1 (34) SOX10/SOX10*: Neural crest stem cell TF, also marks the NCSC-like state (14)
		JunB/*JUNB*: AP-1 transcription factor subunit; possibly negative regulation of the invasive phenotype (62) Fra2/*FOSL2*: AP-1 transcription factor subunit; negative regulation of the invasive phenotype (63)
	Secretory factors/Extracellular ligands	WNT5A/*WNT5A* (27, 64–66) TNFα/*TNF* (7, 48, 67) TGFβ/*TGFB1* (6, 42, 68, 69) Others: IL-1/*lL1*, IL-6/IL6, CCL2/*CCL2*, MMP-2/*MMP2*, MMP-9/*MMP9*, ANGPT2/*ANGPT2*, IGFBP2/*IGFBP2*, IGFBP6/*IGFBP6* (70)
	Receptors	AXL/*AXL*: Hallmark of the invasive melanoma signature (12, 14, 16, 27, 47, 64, 71) ROR2/*ROR2*: WNT5A receptor (39, 66) NOTCHs/*NOTCH1, NOTCH2, NOTCH3, NOTCH4* (45, 72–74) EGFR/*EGFR* (75) PDGFR/*PDGFRA* (76) CD36/*CD36*: Hallmark of the starved-like melanoma cell (SMC) state (16, 77) NGFR/*NGFR*: Hallmark of the NCSC-like phenotype (14, 16, 54, 78–80) GFRA2/*GFRA2*: Marker of the NCSC-like state (16)
	Epigenetic regulators	BMI1/*BMI1* (81) EZH2/*EZH2* (72)
	Translational factors	eIF2α (phospho Ser51)/*EIF2A*: Upstream regulator of ATF4/*ATF4* eIF4E (phospho Ser209)/*EIF4E*: Upstream regulator of NGFR/*NGFR*
	Others	RXRγ/RXRG: Nuclear receptor, driver of the NCSC-like state (16) AQP1/*AQP1*: Marker of the NCSC-like state (16)

It is worth mentioning that, apart from the conventional two-state system, several additional states, including the NGFR^high^-marked neural crest stem cell (NCSC)-like state and an intermediate (transitory) state, have been proposed (14–16). While the NCSC-like state is also associated with low MITF expression, similar to the invasive/mesenchymal-like state, it is marked with high levels of NGFR, SOX10, AQP1, GFRA2, and RXRγ. This state of cells is highly enriched upon therapy and is thought to be a more drug-resistant version of the invasive state (14, 16). The intermediate state likely occurs when melanoma cells are switching between the proliferative and the invasive phenotypes. This transitory state is marked with concurrent enrichment of neural crest and pigmentation-associated gene sets and exhibit intermediate MITF activity (14–16). Notably, CD36 marks a unique population of this intermediate phenotype, termed the starved-like melanoma cells (SMCs) (16). These cells exhibit an altered metabolic gene signature and have increased tolerance to nutrient starvation and targeted therapy agents (3, 16, 77).

Nevertheless, the invasiveness-associated genes/proteins highlight the activation of multiple pathways that play central roles in cancer cell stress adaptation (**Table 1**). Melanoma switching to an “invasive” phenotype can be experimentally driven by environmental stress signals summarized in **Table 2**. For example, melanoma cells cultured in conditions such as hypoxia (8, 55, 101), glucose and glutamine starvation (9, 57), presence of inflammatory cytokines such as TNF-α (7, 48, 86) and TGFβ (102), ultimately switch to an invasive state. In addition, chronic exposure of cultured melanoma cells to BRAF and/or MEK inhibitors (54, 78) and melanocytic antigen-specific T cells (103) promotes the invasive switch, a phenotype that can be recapitulated *in vivo* (7, 104, 105). Here, we highlight essential signaling pathways that have been identified to drive melanoma phenotype switching and their roles in different stress conditions.

**Table 2:** Stress-specific pathways driving melanoma phenotype switching.

Conditions	Factors	Master regulators	Signaling pathways/mechanisms
Metabolic stress	Hypoxia	HIF1α	HIF1α → BHLHE40/BHLHB2 (⊣MITF) → dedifferentiation (8, 55) HIF1α → WNT5A-ROR2 → invasion (39, 66, 82) Akt → NF-κB (+HIF1α) → Notch1 → phenotype switching (83, 84)
	Nutrient starvation	p-eIF2α ATF4	p-eIF2α (⊣eIF2B) → ATF4 → AXL → invasion (9, 57) p-eIF2α (⊣eIF2B) → ATF4 (⊣MITF) → dedifferentiation (9, 57)
	Oxidative stress	NRF2	NRF2 → ATF4 (⊣MITF) → dedifferentiation, inflammation (85)
Inflammation & cytokines	TNFα	BRN2 AP-1 (c-Jun)	TNFα → BRN2 → phenotype switching (Summarized in **Figure 2**) (86, 87) TNFα → c-Jun → phenotype switching (Summarized in **Figure 2**) (48, 88, 89)
	TGFβ	ATF4 AP-1 (c-Jun) HIF1α	TGFβ → ATF4 (⊣MITF)⊣proliferation, differentiation (90, 91) TGFβ → c-Jun (or JunB) → phenotype switching (Summarized in **Figure 2**) (52) TGFβ (⊣PHD2) → HIF1α → phenotype switching (92)
	IL-1	NF-κB AP-1	IL-1 → phospho-IκB → NF-κB → phenotype switching (93) IL-1 → JNK → AP-1 (c-Jun) → phenotype switching (94) IL-1 → MMP-9 → invasion (95, 96) IL-1 (⊣MITF) → dedifferentiation (97)
	IL-6	WNT5A CA-IX	IL-6 → MAPK → WNT5A → phenotype switching (98) IL-6 → CA-IX → phenotype switching (99, 100)

Extracellular Ligands.

Receptors.

Transcription factors.

Transcriptional repressors.

Epigenetic regulators.

Translational factors.

Others.

### Pan-Signal Inducible Signaling Networks

#### The BRN2 Signaling

**Figure 2:**
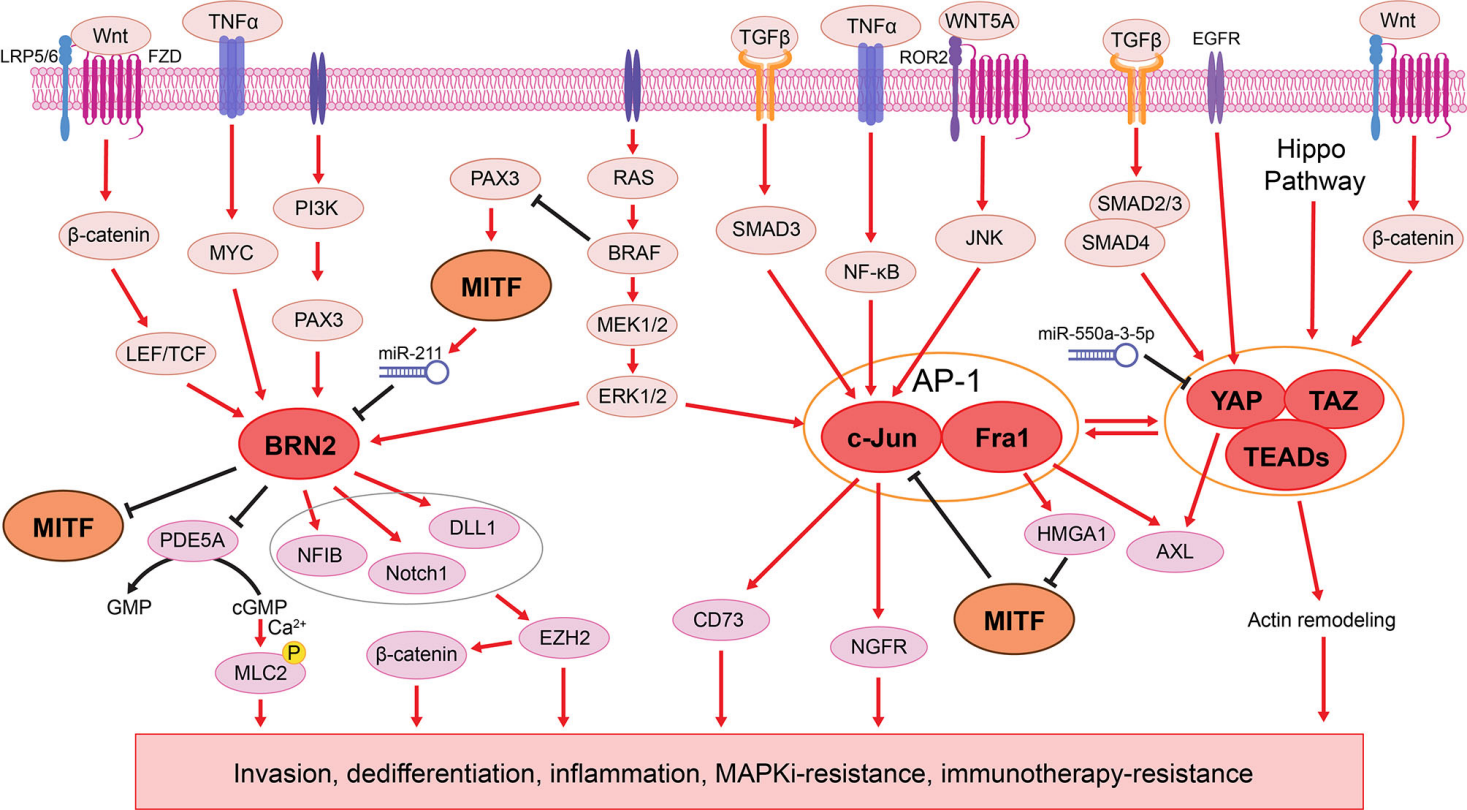
The BRN2 and AP1/TEADs transcriptional networks in melanoma phenotype switching. Left: BRN2 signaling is activated by various pathways, including RAS/RAF/MAPK (106, 107), PI3K/PAX3 (108, 109), Wnt/β-catenin (110), and TNFα/MYC (111, 112) pathways. BRN2 mediates melanoma cell dedifferentiation by inhibiting *MITF* transcription (41), while MITF in turn represses BRN2 through miR-211 (44*)*. BRN2 promotes melanoma cell invasion through transcriptional repression of the PDE5A, resulting in accumulated levels of cGMP and ultimately increased cell contractility (106, 107). BRN2 also induces NFIB (113), Notch1, and DLL1 (45), which together promote melanoma phenotype switching through EZH2 (72, 113) and subsequent activation of the WNT/β-catenin signaling (113, 114). Middle: The AP-1 complex composed of c-Jun and Fra1 is activated downstream of the MAPK pathway (49, 115, 116) and several extracellular ligands, such as TGFβ, TNFα and WNT5A (48, 82, 117–120). AP-1 suppresses MITF through the Fra1 transcriptional target HMGA1 (53), while MITF binds to the *JUN* promoter and blocks its transcription (48, 50). AP-1 also upregulates the expression of AXL (48, 53), NGFR (48), and CD73 (104), driving phenotype switching to the invasive and therapy resistant state (49, 121). Right: Downstream of Hippo, TGFβ, EGFR, and Wnt/β-catenin pathways (122), the YAP/TAZ-TEAD complex cooperates with AP-1 to drive melanoma phenotype switching and therapy resistance through AXL and actin remodeling (123–128).

BRN2 was one of the earliest transcription factors identified as a master regulator of melanoma invasion and metastasis (41–43), with its role in melanoma plasticity comprehensively demonstrated [reviewed by Fane et al. (43)]. Mutually exclusive expression of MITF and BRN2 was identified in patient melanomas and xenografts (41, 42). Mechanistically, BRN2 directly binds to the *MITF* promoter to repress its transcription (41), while increased MITF activity represses BRN2 through miR-211, which is derived from the MITF-target gene *TRPM1* (**Figure 2**) (44). It is tempting to speculate that a reciprocal regulation of BRN2 and MITF expression enables a swift switch between proliferative and invasive phenotypes, which is ultimately required for seeding and outgrowth of melanoma cells at secondary metastatic sites. BRN2 is generally accepted as a pan-signal inducible driver of phenotype switching, activated by coordinated intrinsic oncogene-driven and extracellular factor-induced signaling networks, including the RAS/RAF/MAPK (106, 107), PI3K/PAX3 (108, 109), Wnt/β-catenin (110), and TNFα/MYC (111, 112) pathways (**Figure 2**). For example, in oncogenic BRAF-driven melanomas, BRN2 expression is elevated through hyperactivated MAPK signaling, which transcriptionally represses the cGMP-specific phosphodiesterase PDE5A (106, 107). Consequently, accumulated cGMP leads to an increase in cytosolic calcium ions, which stimulates myosin light chain 2 (MLC2) phosphorylation, thereby inducing contractility and promoting invasion (106, 107). In addition, BRN2 and MITF activate and repress the NOTCH pathway, respectively (45, 129, 130). Activated NOTCH signaling subsequently drives melanoma dedifferentiation and invasion through the epigenetic regulator EZH2 (72, 113) (**Figure 2**).

#### The AP-1 and TEAD Transcription Factor Family

To better understand transcriptional regulators that drive melanoma phenotype switching, Verfaillie et al. found that the invasive gene signature (12, 26, 54, 64) was enriched for AP-1 and TEAD motifs, indicating an AP-1/TEAD-governed transcriptional landscape underpinning the invasive state (25).

AP-1 is a dimeric transcription factor composed of proteins belonging to the Jun (c-Jun, JunB, and JunD), Fos (c-Fos, FosB, Fra1, and Fra2), and closely related transcription factor (ATF2, LRF1/ATF3 and B-ATF) subfamilies (117). AP-1 is activated by both intrinsic oncogene-driven and extrinsic stress-driven MAPK pathway hyperactivation (49, 115, 116), and other stimuli such as inflammatory cytokines and stress inducers (**Figure 2**) (48, 117, 118). For example, the WNT5A/β-catenin-mediated non-canonical Wnt signaling, a well-known driver of melanoma phenotype switching, acts directly upstream of AP-1 (82, 118–120). In response to a variety of environmental stimuli, the extracellular ligand WNT5A interacts with ROR2 and Frizzled (Fzd)-family receptors, resulting in AP-1 activation (82, 118–120) and subsequently promotes melanoma cell invasion (65, 120). In addition, the AP-1 family member c-Jun is highly active in melanoma cells, and its expression is negatively regulated by MITF, which binds to the *JUN* promoter and blocks its transcription (48, 50). In response to inflammation, TNFα induces the expression of c-Jun, when MITF is suppressed, leading to a switch to the invasive state (48). Hyperactivation of MAPK signaling in BRAF^V600E^-mutant melanoma cells leads to increased c-Jun expression, resulting in mesenchymal-like phenotype and resistance to BRAF/MEK inhibitors (MAPKi) (49). MAPKi-sensitive cell lines develop adaptive resistance through *SPROUTY4* downregulation, which leads to increased abundance of c-Jun and a subsequent switch to the mesenchymal-like and drug-resistant state (49). Another AP-1 family member, Fra1 (*FOSL1*), downregulates MITF through its transcriptional target, the chromatin modifier HMGA1, and induces the expression of AXL, driving melanoma cells to the MITF^low^/AXL^high^ state (53). Notably, while c-Jun and Fra1 promote the melanoma switch to the invasive phenotype, other AP-1 transcription factors, such as Fra2 (63) and perhaps JunB (62) inhibit this switch. These studies indicate that AP-1 mediates plasticity by the differing composition of its subunits, similar to the aforementioned ZEB2/ZEB1 and LEF1/TCF4 switch.

Although regulated downstream of various oncogenic pathways, including Wnt, TGFβ, and EGFR signaling, the TEAD family of transcription factors (TEAD1-4) are best-known as final effectors of the Hippo pathway (122). Upon activation, YAP/TAZ translocate into the nucleus and bind to TEADs to promote transcriptional programs (123). Numerous studies demonstrate a cooperative transcriptional mechanism between AP-1 and TEADs (123–125). For example, YAP/TAZ/TEAD-mediated *FOS* transcription increases AP-1 activity, which in turn contributes to the expression of YAP/TAZ downstream target genes (123). In addition, a significant overlap in the AP-1- and TEAD-mediated gene signatures is observed in invasive melanomas (25). Mechanistically, AXL is identified as a direct transcriptional target of YAP (126). Overexpression of YAP drives melanoma cell invasion and metastasis through induction of an invasive gene signature (127). Increased nuclear accumulation of YAP/TAZ leads to actin remodeling, which in turn confers BRAF inhibitor resistance in BRAF^V600E^-mutant melanoma cells (**Figure 2**) (128). Simultaneous knockdown of all four TEADs in invasive melanoma cultures results in decreased invasiveness and increased sensitivity to MAPKi (25).

### Metabolic Stress-Induced Plasticity

Limited availability of oxygen (hypoxia) and nutrients remain a challenge for most tumors. Numerous studies have demonstrated that hypoxia (8, 39, 55, 66, 83) and nutrient deprivation (9, 57) promote melanoma phenotype switching to the invasive and dedifferentiated state (**Table 2**). In this state, slowly dividing melanoma cells show lower oxygen and nutrient consumption and are hence more “fit”, that is, able to survive under insufficient oxygenation and nutritional support. At the same time, these invasive cells have a better chance to migrate, and colonize new sites in the body where conditions are more optimal for their survival and growth.

#### HIF1α: Hypoxia-Specific Driver of Phenotype Switching

Mechanistically, hypoxia drives melanoma phenotype switching in a HIF1α-dependent manner (**Table 2**). Hypoxia induces the expression of HIF1α, which regulates the expression of BHLHE40 (8, 55), WNT5A and ROR2 (39, 82), and Notch1 (83, 84). BHLHE40 is a transcriptional suppressor capable of binding to the *MITF* promoter to repress its transcription (8, 55). In response to hypoxia, ROR1-expressing melanomas adopt a ROR2-positive invasive phenotype. The tyrosine kinase receptor ROR2 drives invasion through its interaction with WNT5A and downstream non-canonical Wnt signaling (39, 66). In addition, hypoxia induces Akt hyperactivation, which cooperates with HIF1α to promote Notch1 expression, an effector that drives melanoma phenotype switching (45, 72, 83, 84). In non-melanoma cancers, hypoxia activates TGF-β signaling, which then cooperates with HIF1α to promote invasion (131, 132). Finally, CA-IX is a metalloenzyme which is produced following hypoxic stress. In melanoma, CA-IX has been shown to be acidify the tumor microenvironment and participate in tumor growth, survival, invasion, and metastasis (**Table 2**) (99). CA-IX is druggable, and the inhibitor SLC-0111 has shown efficacy in inhibiting melanoma phenotype switching (**Table 3**) (100).

**Table 3:** Therapeutic agents that target melanoma phenotype switching.

Target signaling	Agent	Description	Efficacy
WNT5A signaling	Anti-Fz5 antibody (120)	Frizzled-5 polyclonal antibody	Decrease melanoma cell invasion *in vitro*
	Gö 6983 (133)	PKC inhibitor	Block WNT5A-mediated inhibition of Melan-A and PAX3 *in vitro*
	C59 (134)	Soluble PORCN inhibitor	Decrease melanoma cell-derived WNT5A secretion Synergize with anti-CTLA-4 immunotherapy *in vivo*
AP-1 signaling	JNK-IN-8 (49)	c-Jun N-Terminal Kinase (JNK) inhibitor	Decrease melanoma cell migration *in vitro* Enhance the efficacy of vemurafenib *in vitro*
BRN2 signaling	DZNep (72)	EZH2 inhibitor	Increase melanosomes and pigmentation in melanoma cells Decrease melanoma cell invasion *in vitro*
	GSK343 (113)	EZH2 inhibitor	Increase MITF expression in melanoma cells Decrease melanoma cell migration *in vitro*
	GSK503 (135)	EZH2 inhibitor	Abolishes metastases formation *in vivo*
	AM404 (111, 136)	Inhibitor blocking NFATc2-DNA binding	Increase melanocytic differentiation markers, decrease BRN2, AXL, EZH2 and EMT markers, and decrease melanoma cell invasion *in vitro* AM404+GSK126 (EZH2i) reverse phenotype switching AM404+GSK126 induce apoptosis and sensitize melanoma cells to MAPKi
AXL^high^ dedifferentiated state	Enapotamab vedotin (AXL-107-MMAE) (137, 138)	Human AXL antibody linked to monomethyl auristatin E (MMAE)	Display potent anti-tumor activity *in vivo* as single agent Synergize with MAPK inhibitors to inhibit tumor growth *in vivo* Synergize with anti-PD-1 immunotherapy *in vivo*
NGFR^high^ NCSC-like state	Ganetespib (103)	HSP90 inhibitor	Decrease NGFR expression in T-cell therapy-resistant cells Restore melanoma cell sensitivity to T cell attack *in vitro* Restore tumor sensitivity to T-cell therapy *in vitro*
	17-AAG (103)	HSP90 inhibitor	Decrease NGFR expression in T-cell therapy-resistant cells Restore melanoma cell sensitivity to T cell attack *in vitro*
	AG-879 (103)	NGFR kinase inhibitor	Restore melanoma cell sensitivity to T cell attack *in vitro*
	HX531 (16)	RXR antagonist	Decrease NCSC-like populations upon BRAF/MEK inhibition Enhance the efficacy of dabrafenib+trametinib *in vivo* Delay resistance to dabrafenib+trametinib *in vivo*
	PF562271 (139)	Focal adhesion kinase (FAK) inhibitor	Decrease NCSC-like (GFRA2^+^) populations upon BRAF/MEK inhibition PF562271+HX531 combination delays the onset of therapy resistance further than HX531 alone *in vivo*
CD36^+^ SMC state (proposed)	Sulfosuccinimidyl oleate (SSO) (140–142)	CD36 inhibitor	Re-sensitize drug-resistant breast cancer cells to lapatinib *in vitro* Block high glucose-induced EMT in renal tubular epithelial cells Ruduce *in vitro* proliferation and *in vivo* growth of colorectal cancer cells.
	Anti-CD36 mAb Clone JC63.1 (140, 143)	CD36-neutralizing antibody	Re-sensitize drug-resistant breast cancer to lapatinib *in vivo* Inhibit metastasis of oral squamous cell carcinomas *in vivo* Reduce the size of metastases of squamous cell carcinomas *in vivo*
	Anti-CD36 mAb Clone FA6.152 (143)	CD36-neutralizing antibody	Inhibit metastasis of oral squamous cell carcinomas *in vivo*
eIF4F complex	Silvestrol (144, 145)	eIF4A RNA helicase inhibitor	Selectively kill melanoma persister cells *in vitro* Inhibit the emergence of persister cells, combined with BRAFi and MEKi Synergistically inhibit melanoma cell growth *in vitro*, combined with BRAFi
	FL3 flavagline (145)	eIF4A inhibitor	Strong reduction of melanoma growth, combined with BRAFi *in vivo*
	4EG inhibitor-1 (4EGI-1) (145)	Inhibitor blocking eIF4E-eIF4G interaction	Selectively kill vemurafenib-resistant melanoma cells *in vitro* Synergize with vemurafenib in inhibit melanoma cell growth *in vitro*
	SEL201 (146)	MNK1/2 kinase inhibitor	Decrease NGFR expression and increase MITF expression *in vitro* Decrease melanoma cell invasion *in vitro* Restore melanoma cell sensitivity to vemurafenib *in vitro* Sensitize melanoma to anti-PD-1 immunotherapy *in vivo*
	eFT508 (146)	MNK1/2 inhibitor	Sensitize melanoma to anti-PD-1 immunotherapy *in vivo*
Others	SLC-0111 (100)	CA-IX inhibitor	Revert mesenchymal stem cell (MSC)-mediated melanoma phenotype switching and vemurafenib resistance *in vitro*

#### ATF4 in Nutrient Deprivation-Induced Phenotype Switching

**Figure 3:**
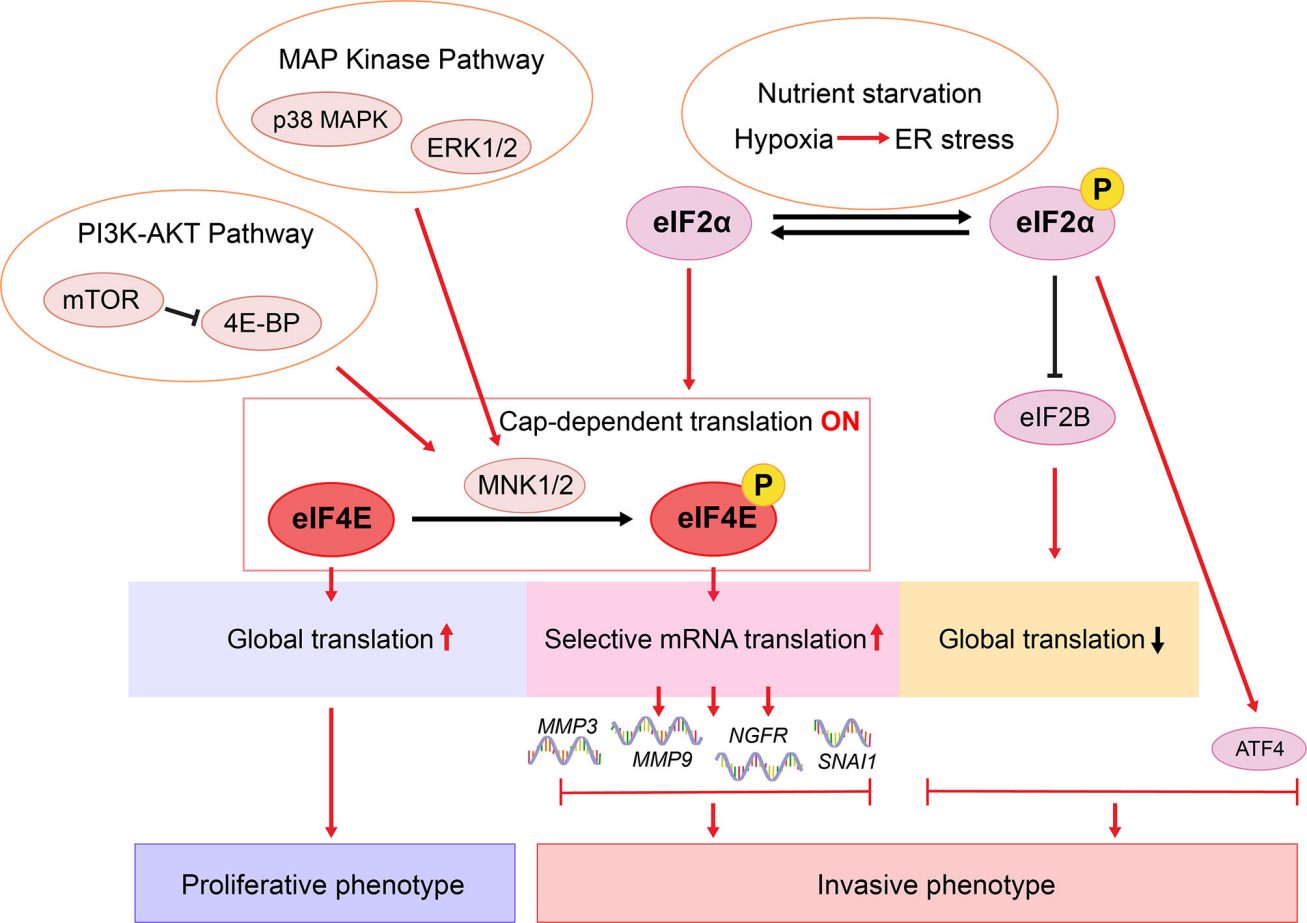
Proposed translational regulation of melanoma phenotype switching. In response to environmental stress such as nutrient starvation, p-eIF2α mediates ISR and inhibits eIF2B activity, which diminishes global translation whilst increasing the translation of ATF4 (9, 57, 147, 148). ATF4 cooperates with the p-eIF2α-mediated translational reprogramming to drive phenotype switching (9, 57). In a nutrient sufficient environment, eIF2α is not phosphorylated and cap-dependent translation is on. Efficient global translation enables melanoma cells to sustain a proliferative state (9). Hyperactivated MAPK and PI3K-AKT pathways lead to MNK1/2-mediated eIF4E phosphorylation, enhancing the translation of a selected subset of mRNAs, including *NGFR*, *MMP3*, *MMP9*, and *SNAI1 (*146, 149, 150). Consequently, these oncogenes promote melanoma cell invasion, metastasis, and therapy resistance (37, 70, 146, 149, 151, 152).

Glucose and glutamine starvation drives melanoma phenotype switching through the ISR-associated transcription factor ATF4 (**Figure 3** and **Table 2**) (9, 57). Nutrient limitation leads to the phosphorylation of eIF2α (p-eIF2α), a hallmark of ISR. P-eIF2α inhibits eIF2B activity, which diminishes global translation whilst increasing the translation of select mRNAs, including ATF4, to mitigate the effects of nutrient deprivation (9, 57, 147, 148). ATF4 activates *AXL* and represses *MITF* transcription, which subsequently slows down cell proliferation and drives a switch to the invasive phenotype (9). Notably, increased ATF4 activity alone is not sufficient to drive phenotype switching. Rather, it cooperates with the p-eIF2α-mediated translational reprogramming (9).

### Inflammation/Cytokine-Induced Plasticity

Inflammation was one of the first microenvironmental triggers identified to drive melanoma dedifferentiation (6, 7, 26). Studies focusing on the invasive switch of melanoma cells within primary tumors have found that stromal cells secrete pro- and anti-inflammatory factors such as TGF-β, TNF-α, MMPs, cytokines and WNT5A into the tumor microenvironment, all of which act to increase invasion and tumor cell dissemination. Curiously, melanoma cells in the invasive state produce pro-inflammatory factors, and in a paracrine manner stimulate proliferative melanomas to switch to the invasive state, a process termed “phenotype cooperativity” [reviewed by *Arozarena & Wellbrock* (13)].

#### TNFα

Tumor necrosis factor alpha (TNFα) is a soluble or membrane pro-inflammatory cytokine produced by macrophages, T-cells, and NK cells. In the case of melanoma, TNFα is involved in many of the mechanisms known to alter plasticity, leading to tumor escape by increasing adhesion molecules such as fibronectin (153). TNFα induces BRN2 signaling (87) and, in line with often mutually exclusive expression of BRN2 and MITF, it has been identified as an inhibitor of MITF and Melan A in 40 different melanoma cell lines (86). Interestingly, MITF expression can modify the inflammatory status of the tumor microenvironment by inhibiting c-Jun and subsequently decreasing TNFα (48). This results in a decrease in myeloid cell recruitment to the tumor, less inflammation and potentially less inflammation associated phenotype switching. These results suggest MITF^low^/c-Jun^high^ melanoma cells enhance phenotype switching by increasing TNFα production. Furthermore, the invasiveness pathway activated by AP-1 can be triggered by inflammatory cytokines from TNF family (88) and AP-1 can positively regulate TNFα production thereby amplifying phenotype switching (**Table 2**) (89). TNFα can also promote tumor cell dissemination by increasing angiotropism (67, 154), the process whereby melanoma cells around blood vessels leads to metastasis without entry into the blood circulation. Tüting’s team highlighted the role of TNFα in the enhancement of endothelial cell sprouting and promoted the pericyte-like expansion of co-cultured melanoma cells along such endothelial outgrowths. Co-culture of melanoma cells with endothelial cells composing the abluminal surface of blood vessels, was demonstrated to induce the expression of genes linked to cancer cell migration (CCL2, ICAM1), cancer progression (TRAF1, SERPINB2) or stem cell properties (PDGFB; CFDP1) (155). These data suggest the interaction between the abluminal surface of endothelial cells and melanoma cells could lead to a melanoma phenotype switch favoring metastasis.

TNFα can have additional unwanted effects on anti-tumor immune responses. First, TNFα can decrease the recognition of tumor cells by melanocytic antigen-specific CD8^+^ T-cells (7) and trigger the death of CD8^+^ T cells (156). Moreover, regulatory T-cells, which are generally considered a bad prognostic factor in melanoma (157), can strongly express TNFR2 on their surface, are the most potent Tregs and can survive longer than the others in the presence of TNFα (158).

#### TGFβ

Transforming growth factor β (TGFβ) is a family of 40 proteins including TGFβ, Activins and Nodal. TGFβ is an anti-inflammatory cytokine important in the control of inflammation, its lack leads to lethal inflammation (159). TGFβ is expressed by melanocytes, negatively regulating their proliferation. However, when melanoma initiates, tumor cells resist the anti-proliferative effects of TGFβ while continually producing it (160). As described in the previous sections, phenotype switching can be induced in a number of ways and it is worth mentioning that TGFβ is involved in most of these mechanisms, such as in the regulation of MITF expression or as a hypoxia-specific driver of phenotype switching. TGFβ is a negative regulator of MITF leading to the shift from a proliferative to an invasive state (90). Furthermore, in triple negative breast cancer, arguably the most therapy resistant breast cancer, TGFβ promotes ATF4 expression which is correlated with a poor prognosis (91, 161). TGFβ has also been associated with the BRN2 invasive phenotype (42, 162), a pathway known to downregulate MITF. Moreover, TGFβ activates the AP-1 pathway leading the production of c-Jun and JunB, respectively in epidermal keratinocytes and dermal fibroblasts (163), known to be involved in the process of phenotype switching and BRAF inhibitor resistance in melanoma (**Table 2**) (49, 51).

As we described above, hypoxia plays a central role in phenotype switching through the expression of HIF1α. TGFβ can trigger the expression of HIF1α by selectively inhibiting PHD2 expression (**Table 2**) (92). However, TGFβ is also directly involved in the process of vascularization by inducing the expression of macrophage inhibitory protein 1 and VEGF (164, 165).

Similar to the negative impact of TNFα on anti-tumor immunity, TGFβ supports tumor immune-evasion responses by decreasing the activity of cytotoxic immune cells such as natural killer cells (166) or CD8^+^ T-cells (167), while favoring the generation of regulatory T-cells (153).

#### Interleukin-1 and Interleukin-6

Interleukin (IL)-1 is a major pro-inflammatory cytokine, mainly produced by macrophages (168), monocytes or neutrophils and can be triggered by the activation of the inflammasome (169). Two isoforms of IL-1 exist, IL-1α and IL-1β, which bind the same receptor IL-1R but are encoded by two different genes. Several reports highlight the important role of IL-1 in melanoma. A high concentration of IL-1β has been detected in the plasma of patients with melanoma compared to healthy donors (170). The consequences of this IL-1 over-production include (1) an increase in the phosphorylation of NF-кB inhibitor (IкB), thus freeing NF-кB known to be involved in melanoma development (93) and (2) activation of stress-activated protein kinase/c-Jun N-terminal kinase (JNK) identified as a key factor in melanoma progression (94). IL-1 is also involved in the production of metalloproteinase 9 (MMP-9) (95), which plays an important role in the remodeling of extracellular matrix and enhancing tumor cell invasiveness (96). Furthermore, the level of circulating MMP-9 has been cited as a good candidate to evaluate the response to BRAF inhibitors in melanoma patients (70). In terms of plasticity, IL-1α and β can downregulate the expression of MITF and melanocytic antigens, favoring melanoma dedifferentiation and phenotype switching (171). Lastly, IL-1β can upregulate HIF1α (97), known to inhibit MITF and increase melanoma invasiveness (55) (**Table 2**).

IL-6 is a pro-inflammatory cytokine family of ten proteins involved in the anti-viral immune response. IL-6 is a key factor in the acute phase of inflammation and participates in the establishment of chronic inflammation. IL-6 is produced by macrophages (172), T-cells, B-cells, and endothelial cells (173). IL-6 binds to the IL-6R to produce acute phase proteins such as inflammatory markers (e.g., serum amiloyd A or complement factors) present in serum during inflammation. On the other hand, IL-6 is involved in the differentiation of Th17 (174), follicular helper T-cells (175), or in the proliferation and survival of CD8 T-cells (176). Similar to IL-1, IL-6 is abundant in the serum of patients with melanoma (177). IL-6 increases the invasiveness and motility of melanoma cells through the MAPK pathway which upregulates WNT5A (98), which as discussed above is a major regulator of phenotype switching.

### Insight Into the Role of Translational Regulation of Cellular Plasticity

In the past decade, studies on melanoma phenotype switching have focused largely on mechanisms involving transcriptional reprograming, through which intracellular cues and extracellular signals are integrated to enable rapid shifts in cell states. In recent years, emerging data suggest that translational reprograming collaborates with epigenetic and metabolic programs to promote phenotypic plasticity of cancers (178). In eukaryotic cells, selective translation is regulated mostly, but not exclusively, at the translation initiation step, through the eIF4F complex and the ternary complex (179). In 2017, Falletta et al. and Ferguson et al. independently showed that phenotype switching is driven by an ISR-dependent translational reprogramming through the p-eIF2α-eIF2B-ATF4 axis (9, 57). This ternary complex-mediated pathway is highly associated with nutrient starvation, resulting in increased ATF4 expression coupled with diminished global mRNA translation (9, 57, 147, 148) (**Figure 3** and **Table 2**). Importantly, while blocking p-eIF2α impairs invasion, without translation reprogramming, increased ATF4 activity alone is not sufficient to drive phenotype switching (9). More recently, Huang et al. demonstrated a mechanism involving activation of the MNK1/2-eIF4E axis and cellular plasticity (146). As a key component of the eIF4F complex, eIF4E regulates cap-dependent mRNA translation initiation. Phosphorylation of eIF4E (p-eIF4E) by kinases MNK1/2 selectively enhances the translation of a subset of mRNAs encoding pro-invasive and pro-survival factors (180, 181). Blocking the MNK1/2-eIF4E axis reversed phenotype switching with impaired invasion, increased expression of MITF, and restored sensitivity to MAPK-targeted and immunotherapies (146). While we showed that the MNK1/2-eIF4E axis promotes the translation of *NGFR*, melanoma phenotype switching and therapy resistance (146), little evidence links NGFR inhibition with a fully reversed switch in melanoma cells (13, 79). These data suggest that more complex translational mechanisms are likely involved in driving melanoma plasticity. For example, in numerous models, p-eIF4E are shown to promote the translation of *MMP3*, *MMP9* and *SNAI1* (149, 150, 180), which are known drivers of phenotype switching (37, 151, 152, 182, 183). Moreover, NODAL is shown to promote the invasive phenotype in melanoma (152) and it is regulated downstream of MNK1 signaling in breast cancer cells (184). Given that previous studies have largely focused on characterizing transcriptome changes between melanoma phenotypes (7–9, 25, 28, 57), it will be important to carry out high-throughput translatomic studies using pre-defined models of melanoma cell phenotype switching.

## Melanoma Plasticity Contributes to Therapy Resistance

### Phenotype Switching and MAPK-Targeted Therapy Resistance

**Figure 4:**
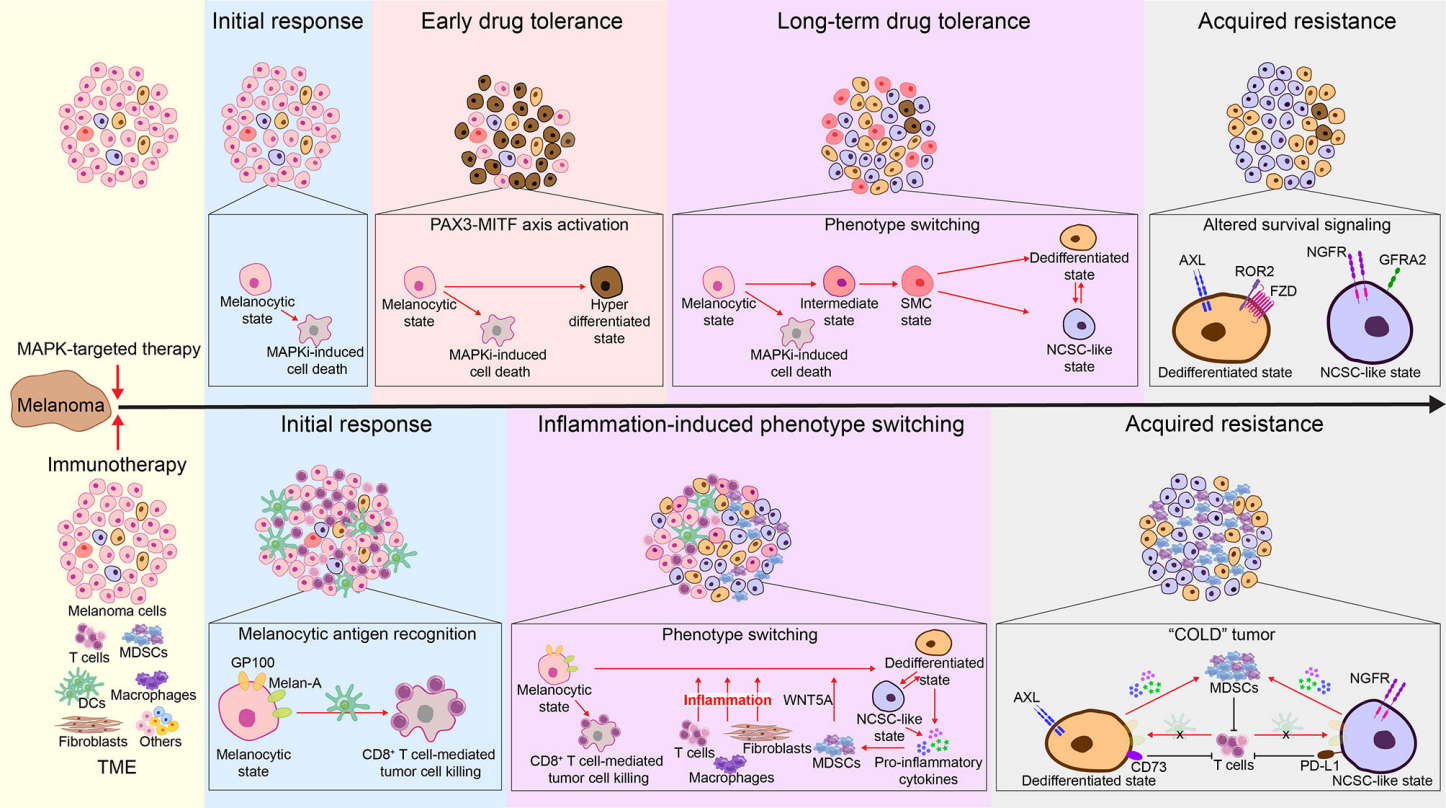
Melanoma phenotype switching in acquired resistance to MAPK-targeted therapy and immunotherapies. Top: During initial response to MAPK-targeted therapy, while melanocytic-state cells are susceptible to MAPKi-induced cell death, a subset of cells switch to a drug-tolerant hyperdifferentiated state mediated by the PAX3-MITF axis. Hyperactivated MITF affords these hyperdifferentiated cells with a survival advantage, enabling them to quickly become the dominant population during the early drug-tolerant phase (16, 185–187). In parallel, remaining cells undergo a fatty acid oxidation-dependent metabolic shift, resulting in the emergence of drug-tolerant CD36^+^ SMC populations (77). These cells undergo a continuous dedifferentiation during prolonged MAPKi treatment resistance (14, 54, 80, 188), resulting in the co-emergence and increase of MITF^low^/AXL^high^ invasive and MITF^low^/NGFR^high^ NCSC-like cells (16). These cells express increased levels of cellular receptors, allowing them to grow, bypassing the MAPK signaling and thereby permitting MAPKi resistance (10, 14, 16, 39, 54, 71, 75, 76, 78, 80, 189, 190). Consequently, at the acquired resistance phase, melanomas show a predominant expression of AXL and NGFR (54, 137). Bottom: During initial response to immunotherapy, activated CD8^+^ T cells recognize melan-A and GP100 antigens expressed on melanocytic-state cells and subsequently eliminate them (7, 31). Meanwhile, immunotherapy- and tumor microenvironment (TME)-induced inflammation drives melanoma phenotype switching, leading to decreased expression of melanocytic antigens and increased levels of pro-inflammatory cytokines (7, 14, 31, 48). Prolonged inflammation leads to increased infiltration of MDSCs (48, 146, 191–195), which further promote phenotype switching *via* secretion of WNT5A (191, 192). In the acquired resistance phase, melanomas are enriched for the MITF^low^/AXL^high^ invasive and MITF^low^/NGFR^high^ NCSC-like populations (103–105). Consequently, these highly dedifferentiated cells escape immune cell recognition (7, 14, 31, 48) and attract high numbers of MDSCs, which further mediate immune suppression (193). In addition, MITF^low^/AXL^high^ invasive cells are associated with high expression of CD73 (104), and MITF^low^/NGFR^high^ NCSC-like cells are found to have increased levels of PD-L1 (105, 196), which ultimately contribute to immunotherapy resistance.

Chronic exposure of cultured melanoma cells to BRAF and/or MEK inhibitors leads to an initial response phase characterized by the induction of MITF and enrichment of MITF^high^ populations, followed by emergence of CD36^+^ SMC populations, which subsequently undergo continuous dedifferentiation, and a final state of acquired resistance marked by a predominance of slow-cycling NCSC-like NGFR^high^ cells (**Figure 4**) (54, 77, 78, 80, 185). In melanoma patients receiving MAPK-targeted therapies, similar patterns of consecutive transcriptional states have also been observed. Notably, during each phase of MAPKi-response and -resistance, different phenotypes (MITF^high^/proliferative, ALX^high^/invasive, and NGFR^high^/NCSC-like) commonly coexist, while predominant population shifting occurs over the treatment course as a result of combined phenotype switching and MAPKi-mediated cell selection (16).

In the initial therapeutic response phase, an immediate activation of the PAX3-MITF-PGC1α axis protects a subset of melanoma cells from MAPKi-induced cell death *via* MITF-mediated survival signaling, resulting in an early drug-tolerant state characterized by an enrichment of MITF^high^ hyperdifferentiated populations (16, 185–187). In parallel, the remaining melanoma cells undergo a fatty acid oxidation-dependent metabolic shift, resulting in a short-term emergence of drug-tolerant CD36^+^ SMC populations (77). The SMC-state cells express medium to low levels of MITF and subsequently undergo dedifferentiation, which allows these slow-cycling cells to persist until the (a) acquisition of new mutations that confer resistance (197), or (b) prolonged MAPKi exposure stabilizes this dedifferentiated state and leads to acquired resistance (14, 54, 80, 188). Notably, activation of ATF4 signaling can be detected in a small group of early MAPKi-tolerant cells, which exhibit a MITF^low^/AXL^high^ invasive-like (dedifferentiated) phenotype (198). Thus, ATF4-mediated phenotype switching might occur early as melanoma cells are adapting to MAPKi therapy. Similarly, the MITF^low^/NGFR^high^ NCSC-like cells also emerge during early MAPKi treatment (16, 139). The MITF^high^ hyperdifferentiated, CD36^+^ SMC, MITF^low^/AXL^high^ dedifferentiated, and MITF^low^/NGFR^high^ NCSC-like populations together make up a reservoir of early MAPKi-persister cells known as minimal residual disease, from which relapse inevitably arises (**Figures 1**, **4**) (16, 139). Following the continued dedifferentiation, MITF^low^/AXL^high^ dedifferentiated and MITF^low^/NGFR^high^ NCSC-like cells increase in number and often co-emerge within the same tumor (16). These cells express increased levels of several cellular receptors, such as AXL, ROR2, PDGFR, EGFR, NGFR, and GFRA2 (10, 14, 16, 39, 54, 71, 75, 76, 78, 80, 189). Mechanistically, these receptors, most being receptor tyrosine kinases, drive alternative survival signaling bypassing the MAPK pathway, thereby permitting MAPKi resistance (71, 189, 190). Consequently, at the acquired resistance and relapse stage, melanomas show a predominant expression of AXL and NGFR, along with an overall trend towards an AXL/AP-1/TEAD-driven gene signature (**Figure 4**) (54, 137). Importantly, as mentioned above, invasive melanoma cells communicate with proliferative melanoma cells through secretory factors to drive their switch to the invasive state. Therefore, an abundance of pre-existing invasive and NCSC-like cells, which are prone to survive initial MAPKi treatment, could cooperate with BRAF/MEK inhibitors to accelerate the development of drug resistance mediated *via* phenotype switching.

### Phenotype Switching and Immunotherapy Resistance

In this last decade, immunotherapies have revolutionized cancer treatment. Immune checkpoint blockade (ICB) targeting the PD-1/PD-L1 and the CTLA-4 axes have become the frontline therapy for patients with metastatic melanoma. In addition, adoptive T-cell transfer therapy is actively being tested in pre-clinical melanoma models. These therapies center on enhancing the function of cytotoxic T cells to improve anti-tumor immune responses (199). Consequently, primary and acquired resistance of melanoma to immunotherapies are linked with mechanisms that ultimately lead to compromised cytotoxic T cell function (200, 201). Hugo et al. identified an innate anti-PD-1 resistance gene signature (IPRES) that is enriched in melanoma samples from anti-PD-1 immunotherapy non-responders (202). IPRES shares similarities with the invasive melanoma gene signature, including *AXL*, *ROR2*, *WNT5A*, *EGFR* and *PDGFRA*, as well as some other phenotype switching-associated genes, such as *TWIST2*, *MMPs*, *ANGPT2*, *IGFBP-6*, *SNAI1, CCL2* (146, 152, 202). In addition to intrinsic resistance, melanoma could also develop acquired resistance to both ICB and adoptive T cell transfer therapy *via* phenotype switching (**Figure 4**) (7, 14, 31, 103, 105). Mechanistically, immunotherapy-induced inflammation drives melanoma dedifferentiation, leading to decreased expression of melanocytic antigens, which impairs T-cell recognition and elimination of tumor cells (7, 14, 31). Moreover, melanoma of the invasive phenotype is associated with increased infiltration of myeloid-derived suppressor cells (MDSCs) (48, 146, 191–195). MDSCs drive melanoma cells to the invasive state, at least in part, *via* their secretion of WNT5A (191, 192), which in turn increases pro-inflammatory cytokine secretion by tumor cells and facilitates MDSC recruitment and function (48, 146). MDSCs mediate immune suppression and ultimately promote immunotherapy resistance (193). In addition, the cell surface enzyme CD73 (5’ ectonucleotidase) is upregulated by AP-1 during therapy-induced phenotype switching (104), which in turn limits the anti-tumoral functions of T cells *via* adenosine receptor signaling (121).

Emerging evidence suggests a link between NGFR^high^ NCSC-like melanoma cells and immune escape of tumors (103, 105). Although the mechanism by which NGFR promotes immunotherapy resistance remains unclear, the BDNF-NGFR axis is suggested to contribute to T-cell therapy resistance (103), and a subset of NGFR-regulated cytokines are linked to immune suppression (146). Notably, NGFR^high^ expressing melanoma cells also showed increased PD-L1 expression (**Figure 4**) (105, 196), with NGFR and PD-L1 protein synthesis both being under translational control *via* the eIF4F complex (146, 203, 204). Again, these studies highlight the importance of examining the translational regulation of melanoma phenotype switching. Finally, it is worth mentioning that, although melanoma phenotype switching is an emerging mechanism underpinning immunotherapy resistance, its prognostic value in predicting responders is debatable. For example, in the case of desmoplastic melanoma where the dedifferentiation phenomenon is high (205), the mutation rate is also important as it leads to the apparition of neoantigens and a better response to anti-PD-1 (206).

## Uveal Melanoma Plasticity and Immune Suppression

Whereas the role of cellular plasticity in cutaneous melanoma has been widely described, phenotype switching in non-skin melanomas, acral and uveal melanomas remains less clear (207). Uveal melanoma (UM) is a malignancy originated from the melanocytes in the uveal tract. The pathogenesis of UM differs from cutaneous melanoma, where its molecular landscape and metastatic outcome presents different challenges (208). UM frequently express mutually exclusive mutations in *GNAQ* and *GNA11*. *GNAQ* and *GNA11* encode G-protein alpha-subunits that mediate signaling downstream from G-protein-coupled receptors, leading to the constitutive activation of diverse signaling pathways such as MAPK and PI3K/AKT/mTOR (209). UM rarely harbour mutations in *BRAF* and is thus not treatable with the BRAF-targeted therapies used in the management of cutaneous melanoma (210, 211). No treatment, including immune-targeted therapies, has shown efficacy in this high fatality ocular cancer. A unique characteristic of UM is the tendency to metastasize to the liver at early stages of the disease, where cells remain dormant until specific signals provided by the primary tumor, or the liver microenvironment, triggers cell proliferation (212).

Studies have been performed to determine potential prognostic factors and biomarkers in UM. In 2006, primary UM were clustered into two main classes related to their gene expression profile (also referred to as mRNA class) (213). Class 1 UM has low or medium metastatic potential, while the Class 2 UM present high metastatic potential with the deletion of chromosome 3. This classification also seems to relate to some characteristics of tumor cell plasticity. Whereas Class 1 UM present a melanocytic/neural crest phenotype, the Class 2 UM are characterized by the downregulated expression of melanocyte-specific and neural crest specification genes (e.g., *TYR, DCT*, *EDNRB*) and upregulated epithelial and cell adhesion markers (e.g., *EMP1/3, CDH1*). Whereas epithelial-derived tumors lose expression of *CDH1* (i.e., E-cadherin) as they gain invasive and metastatic potential, E-cadherin upregulation plays an important role in the dissemination of Class 2 UM. In that context, E-cadherin allows circulating tumour cells to survive once intravasation has been achieved, providing cell-cell interactions so the tumor cells avoid apoptosis (213).

Studies have been performed in recent years to better define the role of cellular plasticity (i.e. EMT) in UM. Repression of a number of transcriptional regulators of EMT, namely ZEB1, TWIST1, and SNAIL, results in decreased UM invasiveness (214). Other critical regulators of cutaneous melanoma phenotype switching are also important in UM. Class 2 UM tumours present with an upregulation of certain proteins and ligands related to the Notch pathway, triggering proliferation and invasion *in vitro* and *in vivo*, thus making it a possible druggable target (215). The expression of IGF-R, c-Fos and c-Jun in patients with Class 2 liver metastasis, indicates a role for plasticity in metastatic UM (216). Additional factors that might have a role in UM plasticity have been explored, such as HIFs and their role in regulating the expression of c-met and C-X-C chemokine receptor type 4 (CXCR4) (217). Moreover, expression of TGF-β and proinflammatory molecules like IL-6 and IL-8 in the liver microenvironment might enhance the survival of UM in the liver (218).

A major signaling pathway that has been proposed to have a pivotal role in UM proliferation and invasion, is the YAP pathway, which lies downstream target of GNAQ and GNA11 aberrant signaling (219). Whereas the reliance of UM on YAP signaling is still debated, in other cancers this signaling pathway has a central role in plasticity (220, 221). The role of YAP/TEADs and AP-1 cooperation was explored in UM tumors lacking expression of LATS1/2 and Kras activation. This study showed that YAP/TEADs and MAPK might cooperate to reinforce each other’s signaling, where AP-1 upregulation or its factors (c-Jun) might have a role in increasing the transcriptional activity of YAP/TEADs (221).

Tumor progression attributed to cancer stem cells (CSCs) has also been explored in UM. CSCs have been described as small clusters of cells that can trigger tumor growth, proliferation and increased metastatic potential. It was found that UM cell lines show upregulation of putative markers of stem-like cells (e.g., CD44, CD133) but are not specific to a subset population of UM cells. The authors suggested UM plasticity might be explained by the neural crest origin of uveal melanocytes rather than presenting a specific hierarchy (222). Nevertheless, other studies showed that expression of nestin, CD166 and NGFR by specific clusters of cells provided a survival advantage, increased metastatic potential and migratory capacity of UM cells (223). Finally, it appears that NGFR also plays an important role in the plasticity and progression of UM. Primary choroidal UM express NGFR, whereas *in vitro* UM cell lines express NGFR when grown in a 3D matrix. In addition, vascular mimicry forming UM cell lines specifically express NGFR, indicating that there might be a possible role of NGFR in UM aggressiveness and resistance to therapy (105, 224). In summary, it is becoming evident that similar to cutaneous melanoma, the phenotypic states of UM will likely be an important mechanism underpinning metastasis. Future work employing single-cell level characterization of UM should enable a deeper understanding of the transitory cell states and phenotypic heterogeneity underlying this high fatality cancer.

## Emerging Strategies to Target Melanoma Plasticity and Their Potential Clinical Impact

Given the growing understanding of the regulatory networks that drive phenotype switching and its role in melanoma metastasis and therapy resistance, an important next step in research is whether this process is targetable and if so, how can we target it pharmacologically? One strategy is to direct melanoma cells towards the more therapy-sensitive proliferative/melanocytic state by blocking or reversing the invasive switch (225). In the past years, several therapeutic agents have been developed and tested (**Table 3**), among which the WNT5A-Protein Kinase C (PKC) pathway was among the first to be targeted (120, 133, 134). In 2002, a polyclonal antibody against the WNT5A receptor Frizzled-5 showed efficacy in decreasing melanoma cell invasion (120). Another early study showed that a pan-PKC inhibitor, Gö 6983, blocked WNT5A-mediated melanoma dedifferentiation *in vitro*, with increased expression of PAX3 and melan-A (133). Moreover, a soluble PORCN inhibitor C59, which functions by blocking WNT5A secretion, showed a synergistic effect with an anti-CTLA-4 antibody *in vivo* (134). Together, these studies provided early evidence that blocking phenotype switching is a promising strategy to sensitize melanoma to standard of care therapies.

More recently, several therapeutic agents that target the transcriptional/epigenetic reprogramming of phenotype switching have been tested. The JNK inhibitor JNK-IN-8, which reduces c-Jun phosphorylation, decreased melanoma cell migration and sensitized melanoma cells to BRAF inhibition (49). Small molecule inhibitors against EZH2, a key effector of the BRN2 signaling, restored differentiation and impaired invasion of melanoma cells *in vitro* (72, 113), and inhibited melanoma growth and metastasis in mouse models (135). Similarly, pharmacological inhibition of NFATc2, a transcription factor that acts upstream of BRN2, drove a melanoma cell switch to a more differentiated state with decreased invasiveness (111, 136). Importantly, combined inhibition of NFATc2 and EZH2 (i.e., AM404+GSK126, respectively) reversed melanoma phenotype switching overnight (136). That drug combination further induced apoptosis in treatment-naïve melanoma cells and restored drug sensitivity in MAPKi-resistant melanoma cells (136). Notably, AM404 alone reversed phenotype switching following a 6-day treatment (111, 136). These observations are in agreement with a paradigm that switching between cell phenotypes is driven by various signals over a prolonged period of time (13), and indicate that co-targeting multiple drivers of phenotype switching might be necessary to achieve optimal therapeutic efficacy.

An alternative strategy is to co-target different cell states of therapy resistance, namely, the SMC state, the dedifferentiated state, and the NCSC-like state. This is feasible due to specific markers for each cellular state (**Figure 1**). For example, enapotamab vedotin (AXL-107-MMAE), was designed to specifically target the AXL^high^ dedifferentiated melanoma cells (137). By eliminating the MITF^low^/AXL^high^ therapy-tolerant and the MITF^high^/AXL^low^ therapy-sensitive populations, respectively, enapotamab vedotin combined with MAPK inhibitors or with an anti-PD-1 antibody, cooperatively inhibited melanoma growth *in vivo* (137, 138). Several agents have shown efficacy targeting the MITF^low^/NGFR^high^ NCSC-like populations, including HSP90 inhibitors (103), an NGFR kinase inhibitor AG-879 (103), an RXR antagonist HX531 (16), and a focal adhesion kinase (FAK) inhibitor PF562271 (139). As anticipated, blocking the NCSC-like state of cells led to enhanced/restored therapy sensitivity (16, 103, 139). The drug-tolerant SMC state is marked by the expression of CD36 (16, 77). It is therefore logical to target this population using CD36 inhibitors or neutralizing antibodies (3). Notably, several CD36-blocking agents have been identified and shown anti-tumor activities in different cancer types, including melanoma (140, 141, 143). Building on the concept that redirecting cell state switching might be necessary to achieve optimal therapeutic efficacy, the Marine group went on to show that melanoma lesions escaping a combination of MAPK-targeted therapy (dabrafenib+trametinib) and NCSC-directed therapy (HX531+PF562271) could be further targeted by ERK inhibitors (139).

Finally, accumulated evidence suggested that the mRNA translation machinery represents a therapeutic opportunity to modulate cancer cell plasticity and overcome therapy resistance (9, 144–146, 178). While agents targeting the eIF2α-eIF2B axis/ternary complex-mediated phenotype switching are yet to be explored, inhibitors targeting different eIF4F components have shown efficacy in preclinical melanoma models. For example, the eIF4A inhibitor silvestrol selectively killed MAPKi-tolerant melanoma cells (144). Combining MAPK inhibitors with an eIF4A inhibitor or with an inhibitor blocking the eIF4E-eIF4G interaction synergistically inhibited melanoma growth (144, 145). MNK1/2 inhibitors, which block eIF4E phosphorylation, decreased melanoma cell invasion, restored MITF expression and repressed NGFR expression in BRAFi-resistant cells, and cooperatively inhibited their growth in combination with vemurafenib (146). Importantly, MNK1/2 inhibitors are capable of sensitizing melanoma to anti-PD-1 immunotherapy in multiple melanoma mouse models (146).

## Conclusions, Perspectives, and Outstanding Questions

Intratumoral heterogeneity and phenotype plasticity is essential for melanoma metastasis and therapy resistance. The phenotype switching model describes a cellular plasticity that enables melanoma cells to adapt to a variety of environmental stress signals including standard of care therapies. Emerging data suggest multiple phenotypic states of melanoma cells, representing a melanocyte differentiation gradient with different sensitivity to therapies, frequently coexisting throughout melanoma progression and throughout anti-cancer treatment (14, 16). At least six distinguished states have been identified, including a MITF^high^ hyperdifferentiated/pigmented state, a MITF^high^ differentiated/melanocytic state, an intermediate state, a CD36^+^ starved-like SMC state, an NGFR^high^ NCSC-like state and an AXL^high^ dedifferentiated state (**Figure 1**) (10, 12–16, 71, 77). Therefore, although most commonly used to describe the proliferative-to-invasive switch, the term “phenotype switching” actually describes cellular plasticity that enables melanoma cells to switch among all the distinct states. With accumulating studies carried out to understand the mechanism underpinning melanoma cell plasticity, it has become clearer to us that the switch between phenotypes is driven by a collaborative reprogramming of the transcriptional, the epigenetic, the translational, and the metabolic regulatory networks (13). In line with the complexity of these networks, blocking multiple drivers of the invasive switch is anticipated, and has been shown, to have better efficacy in reversing phenotype switching than blocking any single driver (111, 136, 139). Similarly, targeting an upstream regulator of the invasive switch has shown superior efficiency than targeting a downstream effector (9). Alternatively, a more direct strategy is to co-target multiple cell states, which has become feasible due to markers defining each cell state (**Figure 1** and **Table 1**). However, a real challenge is, while many state-specific makers have been identified, not all of them are real drivers of phenotype switching. For example, the fatty acid translocase CD36 is a hallmark of the SMC state, which is shown to have an altered metabolic profile (16). It is therefore tempting to speculate that CD36 is a functional driver of the SMC state, perhaps by enabling more efficient uptake of fatty acids and hence confer a selective advantage during therapy (3, 143). However, a recent study suggested that while MAPKi-induced CD36^+^ melanoma cells have increased fatty acid oxidation (FAO), CD36 is not functionally involved in the FAO changes (77). Therefore, inhibitors or neutralizing antibodies against CD36 might not be able to eliminate the SMC population. Rather, a CD36 antibody-drug conjugate, similar to enapotamab vedotin, might be more effective. It is hence important to conduct more studies to distinguish drivers of phenotype switching from melanoma cell state-specific markers.

Another important aspect is the translational control of melanoma plasticity. Whereas the transcriptional, the epigenetic, and the metabolic reprogramming of melanoma phenotype switching have been comprehensively investigated (13), few studies highlight the translational regulation of this process (9, 146). As summarized in **Figure 3**, ATF4 needs to cooperate with reduced energy demand, exhibiting by an inhibition of global mRNA translation, to drive the invasive switch (9). However, blocking the upstream p-eIF2α alone was sufficient to decrease invasion (9), indicating additional regulators downstream of p-eIF2α. Moreover, under nutrient sufficient conditions, cap-dependent global translation is activated, driving melanoma cells to the proliferative state (9, 148). Our recent study suggested, even under such seemingly suitable conditions, melanoma cells could still switch to an invasive state through the MNK1/2-eIF4E axis (146). Notably, phosphorylation of eIF4E doesn’t affect global mRNA translation (149), indicating this invasive switch occurs while maintaining high energy demand (178). Do these two distinct translational regulators lead to different invasive cell states? It is tempting to hypothesize that the p-eIF2α-driven switch leads to a starved-like phenotype, resembling the CD36^+^ SMC state *in vivo* (16, 77), whereas the p-eIF4E-driven switch leads instead to the NGFR^high^ NCSC-like state (14, 16, 79). Further investigation is needed to demonstrate the role of these two translational factors in mediating melanoma phenotype switching.

Finally, uveal melanoma is a complex disease where the role of cell plasticity has not been sufficiently explored. Although exhibiting different genomic mutations from cutaneous melanoma, *GNAQ* and *GNA11* mutations in UM ultimately lead to the hyperactivation of MAPK and PI3K signaling (209). Similar microenvironmental stress conditions such as hypoxia, nutrient limitation, and chronic inflammation are also commonly observed in UM. Thus, UM cells are likely to undergo similar stress-induced phenotypic plasticity. Indeed, many genes that are known to determine invasiveness of cutaneous melanoma cells have also been reported to drive UV progression, such as transcription factors AP-1 and TEADs (219–221), EMT-like markers ZEB1, TWIST1, and SNAIL (214), Notch signaling, and extracellular factors TGF-β, IL-6 and IL-8 (218). These data suggest a potentially significant overlap in plasticity regulators between cutaneous melanoma and UM. Notably, no targeted therapy or immunotherapy has shown efficacy in UM management. Several clinical trials using new molecules, inhibitors alone or in combination are being developed based on preclinical work. MEK inhibitors when combined with dacarbazine or other chemotherapy agents failed to improve progression free survival in UM (226). However, new combination treatments co-targeting MEK with PKC, FAK, GNAQ/11 and other main pathways such as YAP have revealed promising new therapeutic vulnerabilities that can be exploited in a clinical manner (NCT03875820) (227–229). Remarkably, a state-of-the-art immunotherapy agent, Tebentafusp, is showing clinical benefit in patients with advanced UM. Tebentafusp is a molecule that consists in a TCR targeting domain (specific for an antigen of interest) and single-chain variable fragment (scFv) anti-CD3 effector domain. The soluble TCR is designed to bind to GP100, presented by HLA-A*02:01 UM, where the CD3^+^ effector domain will later bind and activate CD3^+^ T cells (230). One might speculate that, similar to immunotherapy resistance in cutaneous melanoma, that the loss of GP100 in UM cells through phenotype switching could lead to Tebentafusp resistance. Therefore, blocking UM phenotype switching is expected to overcome drug resistance, and potentially reduce liver metastasis and sensitize UM cells to future therapies.

## Author Contributions

FH and FS wrote the manuscript. RF wrote sections of the manuscript. FH made figures and tables. SR supervised this study and revised the manuscript. All authors contributed to the article and approved the submitted version.

## Funding

SR is funded by the Canadian Institutes for Health Research (grant PJT-162260) and the Canadian Cancer Society (Emerging Scholar Award Grant # 707140).

## Conflict of Interest

The authors declare that the research was conducted in the absence of any commercial or financial relationships that could be construed as a potential conflict of interest.

## Publisher’s Note

All claims expressed in this article are solely those of the authors and do not necessarily represent those of their affiliated organizations, or those of the publisher, the editors and the reviewers. Any product that may be evaluated in this article, or claim that may be made by its manufacturer, is not guaranteed or endorsed by the publisher.

## References

[1] Jamal-Hanjani M Quezada SA Larkin J Swanton C. Translational Implications of Tumor Heterogeneity. Clin Cancer Res (2015) 21:1258–66. doi: 10.1158/1078-0432.CCR-14-1429 PMC4374162 25770293

[2] Meacham CE Morrison SJ. Tumour Heterogeneity and Cancer Cell Plasticity. Nature (2013) 501:328–37. doi: 10.1038/nature12624 PMC4521623 24048065

[3] Rambow F Marine JC Goding CR. Melanoma Plasticity and Phenotypic Diversity: Therapeutic Barriers and Opportunities. Genes Dev (2019) 33:1295–318. doi: 10.1101/gad.329771.119 PMC6771388 31575676

[4] Sottoriva A Spiteri I Piccirillo SG Touloumis A Collins VP Marioni JC. Intratumor Heterogeneity in Human Glioblastoma Reflects Cancer Evolutionary Dynamics. Proc Natl Acad Sci USA (2013) 110:4009–14. doi: 10.1073/pnas.1219747110 PMC3593922 23412337

[5] Yachida S Jones S Bozic I Antal T Leary R Fu B. Distant Metastasis Occurs Late During the Genetic Evolution of Pancreatic Cancer. Nature (2010) 467:1114–7. doi: 10.1038/nature09515 PMC3148940 20981102

[6] Hoek KS Schlegel NC Brafford P Sucker A Ugurel S Kumar R. Metastatic Potential of Melanomas Defined by Specific Gene Expression Profiles With No BRAF Signature. Pigment Cell Res (2006) 19:290–302. doi: 10.1111/j.1600-0749.2006.00322.x 16827748

[7] Landsberg J Kohlmeyer J Renn M Bald T Rogava M Cron M. Melanomas Resist T-Cell Therapy Through Inflammation-Induced Reversible Dedifferentiation. Nature (2012) 490:412–6. doi: 10.1038/nature11538 23051752

[8] Cheli Y Giuliano S Fenouille N Allegra M Hofman V Hofman P. Hypoxia and MITF Control Metastatic Behaviour in Mouse and Human Melanoma Cells. Oncogene (2012) 31:2461–70. doi: 10.1038/onc.2011.425 21996743

[9] Falletta P Sanchez-Del-Campo L Chauhan J Effern M Kenyon A Kershaw CJ. Translation Reprogramming Is an Evolutionarily Conserved Driver of Phenotypic Plasticity and Therapeutic Resistance in Melanoma. Genes Dev (2017) 31:18–33. doi: 10.1101/gad.290940.116 28096186 PMC5287109

[10] Tirosh I Izar B Prakadan SM Wadsworth MH 2nd Treacy D Trombetta JJ. Dissecting the Multicellular Ecosystem of Metastatic Melanoma by Single-Cell RNA-Seq. Science (2016) 352:189–96. doi: 10.1126/science.aad0501 PMC4944528 27124452

[11] Ennen M Keime C Gambi G Kieny A Coassolo S Thibault-Carpentier C. MITF-High and MITF-Low Cells and a Novel Subpopulation Expressing Genes of Both Cell States Contribute to Intra- and Intertumoral Heterogeneity of Primary Melanoma. Clin Cancer Res (2017) 23:7097–107. doi: 10.1158/1078-0432.CCR-17-0010 28855355

[12] Sensi M Catani M Castellano G Nicolini G Alciato F Tragni G. Human Cutaneous Melanomas Lacking MITF and Melanocyte Differentiation Antigens Express a Functional Axl Receptor Kinase. J Invest Dermatol (2011) 131:2448–57. doi: 10.1038/jid.2011.218 21796150

[13] Arozarena I Wellbrock C. Phenotype Plasticity as Enabler of Melanoma Progression and Therapy Resistance. Nat Rev Cancer (2019) 19:377–91. doi: 10.1038/s41568-019-0154-4 31209265

[14] Tsoi J Robert L Paraiso K Galvan C Sheu KM Lay J. Multi-Stage Differentiation Defines Melanoma Subtypes With Differential Vulnerability to Drug-Induced Iron-Dependent Oxidative Stress. Cancer Cell (2018) 33:890–904.e895. doi: 10.1016/j.ccell.2018.03.017 29657129 PMC5953834

[15] Wouters J Kalender-Atak Z Minnoye L Spanier KI De Waegeneer M Bravo Gonzalez-Blas C. Robust Gene Expression Programs Underlie Recurrent Cell States and Phenotype Switching in Melanoma. Nat Cell Biol (2020) 22:986–98. doi: 10.1038/s41556-020-0547-3 32753671

[16] Rambow F Rogiers A Marin-Bejar O Aibar S Femel J Dewaele M. Toward Minimal Residual Disease-Directed Therapy in Melanoma. Cell (2018) 174:843–55.e819. doi: 10.1016/j.cell.2018.06.025 30017245

[17] Gartner JJ Davis S Wei X Lin JC Trivedi NS Teer JK. Comparative Exome Sequencing of Metastatic Lesions Provides Insights Into the Mutational Progression of Melanoma. BMC Genomics (2012) 13:505. doi: 10.1186/1471-2164-13-505 23006843 PMC3500261

[18] Nikolaev SI Rimoldi D Iseli C Valsesia A Robyr D Gehrig C. Exome Sequencing Identifies Recurrent Somatic MAP2K1 and MAP2K2 Mutations in Melanoma. Nat Genet (2011) 44:133–9. doi: 10.1038/ng.1026 22197931

[19] Sanborn JZ Chung J Purdom E Wang NJ Kakavand H Wilmott JS. Phylogenetic Analyses of Melanoma Reveal Complex Patterns of Metastatic Dissemination. Proc Natl Acad Sci USA (2015) 112:10995–1000. doi: 10.1073/pnas.1508074112 PMC4568214 26286987

[20] Shain AH Bastian BC. From Melanocytes to Melanomas. Nat Rev Cancer (2016) 16:345–58. doi: 10.1038/nrc.2016.37 27125352

[21] Winnepenninckx V Lazar V Michiels S Dessen P Stas M Alonso SR. Gene Expression Profiling of Primary Cutaneous Melanoma and Clinical Outcome. J Natl Cancer Inst (2006) 98:472–82. doi: 10.1093/jnci/djj103 16595783

[22] Hoek KS Goding CR. Cancer Stem Cells Versus Phenotype-Switching in Melanoma. Pigment Cell Melanoma Res (2010) 23:746–59. doi: 10.1111/j.1755-148X.2010.00757.x 20726948

[23] Quintana E Shackleton M Foster HR Fullen DR Sabel MS Johnson TM. Phenotypic Heterogeneity Among Tumorigenic Melanoma Cells From Patients That Is Reversible and Not Hierarchically Organized. Cancer Cell (2010) 18:510–23. doi: 10.1016/j.ccr.2010.10.012 PMC3031091 21075313

[24] Quintana E Shackleton M Foster HR Fullen DR Sabel MS Johnson TM. Efficient Tumour Formation by Single Human Melanoma Cells. Nature (2008) 456:593–8. doi: 10.1038/nature07567 PMC2597380 19052619

[25] Verfaillie A Imrichova H Atak ZK Dewaele M Rambow F Hulselmans G. Decoding the Regulatory Landscape of Melanoma Reveals TEADS as Regulators of the Invasive Cell State. Nat Commun (2015) 6:6683. doi: 10.1038/ncomms7683 25865119 PMC4403341

[26] Hoek KS Eichhoff OM Schlegel NC Dobbeling U Kobert N Schaerer L. In Vivo Switching of Human Melanoma Cells Between Proliferative and Invasive States. Cancer Res (2008) 68:650–6. doi: 10.1158/0008-5472.CAN-07-2491 18245463

[27] Bittner M Meltzer P Chen Y Jiang Y Seftor E Hendrix M. Molecular Classification of Cutaneous Malignant Melanoma by Gene Expression Profiling. Nature (2000) 406:536–40. doi: 10.1038/35020115 10952317

[28] Kim IS Heilmann S Kansler ER Zhang Y Zimmer M Ratnakumar K. Microenvironment-Derived Factors Driving Metastatic Plasticity in Melanoma. Nat Commun (2017) 8:14343. doi: 10.1038/ncomms14343 28181494 PMC5309794

[29] Psaila B Lyden D. The Metastatic Niche: Adapting the Foreign Soil. Nat Rev Cancer (2009) 9:285–93. doi: 10.1038/nrc2621 PMC3682494 19308068

[30] Rambow F Job B Petit V Gesbert F Delmas V Seberg H. New Functional Signatures for Understanding Melanoma Biology From Tumor Cell Lineage-Specific Analysis. Cell Rep (2015) 13:840–53. doi: 10.1016/j.celrep.2015.09.037 PMC5970542 26489459

[31] Mehta A Kim YJ Robert L Tsoi J Comin-Anduix B Berent-Maoz B. Immunotherapy Resistance by Inflammation-Induced Dedifferentiation. Cancer Discov (2018) 8:935–43. doi: 10.1158/2159-8290.CD-17-1178 PMC6076867 29899062

[32] Shakhova O Cheng P Mishra PJ Zingg D Schaefer SM Debbache J. Antagonistic Cross-Regulation Between Sox9 and Sox10 Controls an Anti-Tumorigenic Program in Melanoma. PloS Genet (2015) 11:e1004877. doi: 10.1371/journal.pgen.1004877 25629959 PMC4309598

[33] Cheng PF Shakhova O Widmer DS Eichhoff OM Zingg D Frommel SC. Methylation-Dependent SOX9 Expression Mediates Invasion in Human Melanoma Cells and Is a Negative Prognostic Factor in Advanced Melanoma. Genome Biol (2015) 16:42. doi: 10.1186/s13059-015-0594-4 25885555 PMC4378455

[34] Eichhoff OM Weeraratna A Zipser MC Denat L Widmer DS Xu M. Differential LEF1 and TCF4 Expression Is Involved in Melanoma Cell Phenotype Switching. Pigment Cell Melanoma Res (2011) 24:631–42. doi: 10.1111/j.1755-148X.2011.00871.x 21599871

[35] Denecker G Vandamme N Akay O Koludrovic D Taminau J Lemeire K. Identification of a ZEB2-MITF-ZEB1 Transcriptional Network That Controls Melanogenesis and Melanoma Progression. Cell Death Differ (2014) 21:1250–61. doi: 10.1038/cdd.2014.44 PMC4085532 24769727

[36] Richard G Dalle S Monet MA Ligier M Boespflug A Pommier RM. ZEB1-Mediated Melanoma Cell Plasticity Enhances Resistance to MAPK Inhibitors. EMBO Mol Med (2016) 8:1143–61. doi: 10.15252/emmm.201505971 PMC5048365 27596438

[37] Caramel J Papadogeorgakis E Hill L Browne GJ Richard G Wierinckx A. A Switch in the Expression of Embryonic EMT-Inducers Drives the Development of Malignant Melanoma. Cancer Cell (2013) 24:466–80. doi: 10.1016/j.ccr.2013.08.018 24075834

[38] Siena ADD Placa JR Araujo LF de B II Peronni K Molfetta G. Whole Transcriptome Analysis Reveals Correlation of Long Noncoding RNA ZEB1-AS1 With Invasive Profile in Melanoma. Sci Rep (2019) 9:11350. doi: 10.1038/s41598-019-47363-6 31383874 PMC6683136

[39] O'Connell MP Marchbank K Webster MR Valiga AA Kaur A Vultur A. Hypoxia Induces Phenotypic Plasticity and Therapy Resistance in Melanoma via the Tyrosine Kinase Receptors ROR1 and ROR2. Cancer Discov (2013) 3:1378–93. doi: 10.1158/2159-8290.CD-13-0005 PMC3918498 24104062

[40] Hou L Pavan WJ. Transcriptional and Signaling Regulation in Neural Crest Stem Cell-Derived Melanocyte Development: Do All Roads Lead to Mitf? Cell Res (2008) 18:1163–76. doi: 10.1038/cr.2008.303 19002157

[41] Goodall J Carreira S Denat L Kobi D Davidson I Nuciforo P. Brn-2 Represses Microphthalmia-Associated Transcription Factor Expression and Marks a Distinct Subpopulation of Microphthalmia-Associated Transcription Factor-Negative Melanoma Cells. Cancer Res (2008) 68:7788–94. doi: 10.1158/0008-5472.CAN-08-1053 18829533

[42] Pinner S Jordan P Sharrock K Bazley L Collinson L Marais R. Intravital Imaging Reveals Transient Changes in Pigment Production and Brn2 Expression During Metastatic Melanoma Dissemination. Cancer Res (2009) 69:7969–77. doi: 10.1158/0008-5472.CAN-09-0781 PMC2763120 19826052

[43] Fane ME Chhabra Y Smith AG Sturm RA. BRN2, a POUerful Driver of Melanoma Phenotype Switching and Metastasis. Pigment Cell Melanoma Res (2019) 32:9–24. doi: 10.1111/pcmr.12710 29781575

[44] Boyle GM Woods SL Bonazzi VF Stark MS Hacker E Aoude LG. Melanoma Cell Invasiveness Is Regulated by miR-211 Suppression of the BRN2 Transcription Factor. Pigment Cell Melanoma Res (2011) 24:525–37. doi: 10.1111/j.1755-148X.2011.00849.x 21435193

[45] Thurber AE Douglas G Sturm EC Zabierowski SE Smit DJ Ramakrishnan SN. Inverse Expression States of the BRN2 and MITF Transcription Factors in Melanoma Spheres and Tumour Xenografts Regulate the NOTCH Pathway. Oncogene (2011) 30:3036–48. doi: 10.1038/onc.2011.33 PMC3591523 21358674

[46] Simmons JL Pierce CJ Al-Ejeh F Boyle GM. MITF and BRN2 Contribute to Metastatic Growth After Dissemination of Melanoma. Sci Rep (2017) 7:10909. doi: 10.1038/s41598-017-11366-y 28883623 PMC5589904

[47] Smith MP Rana S Ferguson J Rowling EJ Flaherty KT Wargo JA. A PAX3/BRN2 Rheostat Controls the Dynamics of BRAF Mediated MITF Regulation in MITF(high) /AXL(low) Melanoma. Pigment Cell Melanoma Res (2019) 32:280–91. doi: 10.1111/pcmr.12741 PMC6392120 30277012

[48] Riesenberg S Groetchen A Siddaway R Bald T Reinhardt J Smorra D. MITF and C-Jun Antagonism Interconnects Melanoma Dedifferentiation With Pro-Inflammatory Cytokine Responsiveness and Myeloid Cell Recruitment. Nat Commun (2015) 6:8755. doi: 10.1038/ncomms9755 26530832 PMC4659938

[49] Ramsdale R Jorissen RN Li FZ Al-Obaidi S Ward T Sheppard KE. The Transcription Cofactor C-JUN Mediates Phenotype Switching and BRAF Inhibitor Resistance in Melanoma. Sci Signal (2015) 8:ra82. doi: 10.1126/scisignal.aab1111 26286024

[50] Kappelmann M Bosserhoff A Kuphal S. AP-1/C-Jun Transcription Factors: Regulation and Function in Malignant Melanoma. Eur J Cell Biol (2014) 93:76–81. doi: 10.1016/j.ejcb.2013.10.003 24315690

[51] Gong C Shen J Fang Z Qiao L Feng R Lin X. Abnormally Expressed JunB Transactivated by IL-6/STAT3 Signaling Promotes Uveal Melanoma Aggressiveness via Epithelial-Mesenchymal Transition. Biosci Rep (2018) 38(4):BSR20180532. doi: 10.1042/BSR20180532 PMC6028753 29899166

[52] Verrecchia F Tacheau C Schorpp-Kistner M Angel P Mauviel A. Induction of the AP-1 Members C-Jun and JunB by TGF-Beta/Smad Suppresses Early Smad-Driven Gene Activation. Oncogene (2001) 20:2205–11. doi: 10.1038/sj.onc.1204347 11402315

[53] Maurus K Hufnagel A Geiger F Graf S Berking C Heinemann A. The AP-1 Transcription Factor FOSL1 Causes Melanocyte Reprogramming and Transformation. Oncogene (2017) 36:5110–21. doi: 10.1038/onc.2017.135 28481878

[54] Shaffer SM Dunagin MC Torborg SR Torre EA Emert B Krepler C. Rare Cell Variability and Drug-Induced Reprogramming as a Mode of Cancer Drug Resistance. Nature (2017) 546:431–5. doi: 10.1038/nature22794 PMC5542814 28607484

[55] Feige E Yokoyama S Levy C Khaled M Igras V Lin RJ. Hypoxia-Induced Transcriptional Repression of the Melanoma-Associated Oncogene MITF. Proc Natl Acad Sci USA (2011) 108:E924–933. doi: 10.1073/pnas.1106351108 PMC3203758 21949374

[56] Widmer DS Hoek KS Cheng PF Eichhoff OM Biedermann T Raaijmakers MIG. Hypoxia Contributes to Melanoma Heterogeneity by Triggering HIF1alpha-Dependent Phenotype Switching. J Invest Dermatol (2013) 133:2436–43. doi: 10.1038/jid.2013.115 23474946

[57] Ferguson J Smith M Zudaire I Wellbrock C Arozarena I. Glucose Availability Controls ATF4-Mediated MITF Suppression to Drive Melanoma Cell Growth. Oncotarget (2017) 8:32946–59. doi: 10.18632/oncotarget.16514 PMC5464841 28380427

[58] Strozyk EA Desch A Poeppelmann B Magnolo N Wegener J Huck V. Melanoma-Derived IL-1 Converts Vascular Endothelium to a Proinflammatory and Procoagulatory Phenotype via NFkappaB Activation. Exp Dermatol (2014) 23:670–6. doi: 10.1111/exd.12505 25041487

[59] Ahmed F Haass NK. Microenvironment-Driven Dynamic Heterogeneity and Phenotypic Plasticity as a Mechanism of Melanoma Therapy Resistance. Front Oncol (2018) 8:173. doi: 10.3389/fonc.2018.00173 29881716 PMC5976798

[60] Lin K Baritaki S Militello L Malaponte G Bevelacqua Y Bonavida B. The Role of B-RAF Mutations in Melanoma and the Induction of EMT via Dysregulation of the NF-Kappab/Snail/RKIP/PTEN Circuit. Genes Cancer (2010) 1:409–20. doi: 10.1177/1947601910373795 PMC2933925 20827424

[61] Wardwell-Ozgo J Dogruluk T Gifford A Zhang Y Heffernan TP van Doorn R. HOXA1 Drives Melanoma Tumor Growth and Metastasis and Elicits an Invasion Gene Expression Signature That Prognosticates Clinical Outcome. Oncogene (2014) 33:1017–26. doi: 10.1038/onc.2013.30 PMC3982326 23435427

[62] Nyberg WA He T Sjöstrand M Velasquez-Pulgarin DA Pellé L Covacu R. Melanoma Plasticity Is Controlled by a TRIM28-JUNB Mediated Switch. bioRxiv (2019) 777771. doi: 10.1101/777771

[63] Chen GL Li R Chen XX Wang J Cao S Song R. Fra-2/AP-1 Regulates Melanoma Cell Metastasis by Downregulating Fam212b. Cell Death Differ (2021) 28:1364–78. doi: 10.1038/s41418-020-00660-4 PMC8027635 33188281

[64] Widmer DS Cheng PF Eichhoff OM Belloni BC Zipser MC Schlegel NC. Systematic Classification of Melanoma Cells by Phenotype-Specific Gene Expression Mapping. Pigment Cell Melanoma Res (2012) 25:343–53. doi: 10.1111/j.1755-148X.2012.00986.x 22336146

[65] Webster MR Xu M Kinzler KA Kaur A Appleton J O'Connell MP. Wnt5A Promotes an Adaptive, Senescent-Like Stress Response, While Continuing to Drive Invasion in Melanoma Cells. Pigment Cell Melanoma Res (2015) 28:184–95. doi: 10.1111/pcmr.12330 PMC4333017 25407936

[66] O'Connell MP Fiori JL Xu M Carter AD Frank BP Camilli TC. The Orphan Tyrosine Kinase Receptor, ROR2, Mediates Wnt5A Signaling in Metastatic Melanoma. Oncogene (2010) 29:34–44. doi: 10.1038/onc.2009.305 19802008 PMC2803338

[67] Bald T Quast T Landsberg J Rogava M Glodde N Lopez-Ramos D. Ultraviolet-Radiation-Induced Inflammation Promotes Angiotropism and Metastasis in Melanoma. Nature (2014) 507:109–13. doi: 10.1038/nature13111 24572365

[68] Nishimura EK Suzuki M Igras V Du J Lonning S Miyachi Y. Key Roles for Transforming Growth Factor Beta in Melanocyte Stem Cell Maintenance. Cell Stem Cell (2010) 6:130–40. doi: 10.1016/j.stem.2009.12.010 PMC3437996 20144786

[69] Alexaki VI Javelaud D Van Kempen LC Mohammad KS Dennler S Luciani F. GLI2-Mediated Melanoma Invasion and Metastasis. J Natl Cancer Inst (2010) 102:1148–59. doi: 10.1093/jnci/djq257 PMC2914763 20660365

[70] Salemi R Falzone L Madonna G Polesel J Cina D Mallardo D. MMP-9 as a Candidate Marker of Response to BRAF Inhibitors in Melanoma Patients With BRAF(V600E) Mutation Detected in Circulating-Free DNA. Front Pharmacol (2018) 9:856. doi: 10.3389/fphar.2018.00856 30154717 PMC6102751

[71] Muller J Krijgsman O Tsoi J Robert L Hugo W Song C. Low MITF/AXL Ratio Predicts Early Resistance to Multiple Targeted Drugs in Melanoma. Nat Commun (2014) 5:5712. doi: 10.1038/ncomms6712 25502142 PMC4428333

[72] Manning CS Hooper S Sahai EA. Intravital Imaging of SRF and Notch Signalling Identifies a Key Role for EZH2 in Invasive Melanoma Cells. Oncogene (2015) 34:4320–32. doi: 10.1038/onc.2014.362 PMC4349503 25381824

[73] Golan T Messer AR Amitai-Lange A Melamed Z Ohana R Bell RE. Interactions of Melanoma Cells With Distal Keratinocytes Trigger Metastasis via Notch Signaling Inhibition of MITF. Mol Cell (2015) 59:664–76. doi: 10.1016/j.molcel.2015.06.028 26236014

[74] Bonyadi Rad E Hammerlindl H Wels C Popper U Ravindran Menon D Breiteneder H. Notch4 Signaling Induces a Mesenchymal-Epithelial-Like Transition in Melanoma Cells to Suppress Malignant Behaviors. Cancer Res (2016) 76:1690–7. doi: 10.1158/0008-5472.CAN-15-1722 PMC5167360 26801977

[75] Sun C Wang L Huang S Heynen GJ Prahallad A Robert C. Reversible and Adaptive Resistance to BRAF(V600E) Inhibition in Melanoma. Nature (2014) 508:118–22. doi: 10.1038/nature13121 24670642

[76] Nazarian R Shi H Wang Q Kong X Koya RC Lee H. Melanomas Acquire Resistance to B-RAF(V600E) Inhibition by RTK or N-RAS Upregulation. Nature (2010) 468:973–7. doi: 10.1038/nature09626 PMC3143360 21107323

[77] Aloia A Mullhaupt D Chabbert CD Eberhart T Fluckiger-Mangual S Vukolic A. A Fatty Acid Oxidation-Dependent Metabolic Shift Regulates the Adaptation of BRAF-Mutated Melanoma to MAPK Inhibitors. Clin Cancer Res (2019) 25:6852–67. doi: 10.1158/1078-0432.CCR-19-0253 PMC6906212 31375515

[78] Fallahi-Sichani M Becker V Izar B Baker GJ Lin JR Boswell SA. Adaptive Resistance of Melanoma Cells to RAF Inhibition via Reversible Induction of a Slowly Dividing De-Differentiated State. Mol Syst Biol (2017) 13:905. doi: 10.15252/msb.20166796 28069687 PMC5248573

[79] Restivo G Diener J Cheng PF Kiowski G Bonalli M Biedermann T. Low Neurotrophin Receptor CD271 Regulates Phenotype Switching in Melanoma. Nat Commun (2017) 8:1988. doi: 10.1038/s41467-017-01573-6 29215016 PMC5719420

[80] Su Y Wei W Robert L Xue M Tsoi J Garcia-Diaz A. Single-Cell Analysis Resolves the Cell State Transition and Signaling Dynamics Associated With Melanoma Drug-Induced Resistance. Proc Natl Acad Sci USA (2017) 114:13679–84. doi: 10.1073/pnas.1712064115 PMC5748184 29229836

[81] Ferretti R Bhutkar A McNamara MC Lees JA. BMI1 Induces an Invasive Signature in Melanoma That Promotes Metastasis and Chemoresistance. Genes Dev (2016) 30:18–33. doi: 10.1101/gad.267757.115 26679841 PMC4701976

[82] Wright TM Rathmell WK. Identification of Ror2 as a Hypoxia-Inducible Factor Target in Von Hippel-Lindau-Associated Renal Cell Carcinoma. J Biol Chem (2010) 285:12916–24. doi: 10.1074/jbc.M109.073924 PMC2857057 20185829

[83] Bedogni B Warneke JA Nickoloff BJ Giaccia AJ Powell MB. Notch1 is an Effector of Akt and Hypoxia in Melanoma Development. J Clin Invest (2008) 118:3660–70. doi: 10.1172/JCI36157 PMC2567838 18924608

[84] Bedogni B Welford SM Cassarino DS Nickoloff BJ Giaccia AJ Powell MB. The Hypoxic Microenvironment of the Skin Contributes to Akt-Mediated Melanocyte Transformation. Cancer Cell (2005) 8:443–54. doi: 10.1016/j.ccr.2005.11.005 16338658

[85] Jessen C Kress JKC Baluapuri A Hufnagel A Schmitz W Kneitz S. The Transcription Factor NRF2 Enhances Melanoma Malignancy by Blocking Differentiation and Inducing COX2 Expression. Oncogene (2020) 39:6841–55. doi: 10.1038/s41388-020-01477-8 PMC7605435 32978520

[86] Lim SY Alavi S Ming Z Shklovskaya E Fung C Stewart A. Melanoma Cell State-Specific Responses to TNFalpha. Biomedicines (2021) 9(6):605. doi: 10.3390/biomedicines9060605 34073253 PMC8230114

[87] Hamm M Sohier P Petit V Raymond JH Delmas V Le Coz M. BRN2 Is a Non-Canonical Melanoma Tumor-Suppressor. Nat Commun (2021) 12:3707. doi: 10.1038/s41467-021-23973-5 34140478 PMC8211827

[88] Kyriakis JM. Activation of the AP-1 Transcription Factor by Inflammatory Cytokines of the TNF Family. Gene Expr (1999) 7:217–31. PMC6174675 10440223

[89] Qiao Y He H Jonsson P Sinha I Zhao C Dahlman-Wright K. AP-1 Is a Key Regulator of Proinflammatory Cytokine TNFalpha-Mediated Triple-Negative Breast Cancer Progression. J Biol Chem (2016) 291:5068–79. doi: 10.1074/jbc.M115.702571 PMC4777842 26792858

[90] Pierrat MJ Marsaud V Mauviel A Javelaud D. Expression of Microphthalmia-Associated Transcription Factor (MITF), Which Is Critical for Melanoma Progression, is Inhibited by Both Transcription Factor GLI2 and Transforming Growth Factor-Beta. J Biol Chem (2012) 287:17996–8004. doi: 10.1074/jbc.M112.358341 PMC3365743 22496449

[91] Gonzalez-Gonzalez A Munoz-Muela E Marchal JA Cara FE Molina MP Cruz-Lozano M. Activating Transcription Factor 4 Modulates TGFbeta-Induced Aggressiveness in Triple-Negative Breast Cancer via SMAD2/3/4 and Mtorc2 Signaling. Clin Cancer Res (2018) 24:5697–709. doi: 10.1158/1078-0432.CCR-17-3125 30012564

[92] McMahon S Charbonneau M Grandmont S Richard DE Dubois CM. Transforming Growth Factor Beta1 Induces Hypoxia-Inducible Factor-1 Stabilization Through Selective Inhibition of PHD2 Expression. J Biol Chem (2006) 281:24171–81. doi: 10.1074/jbc.M604507200 16815840

[93] Huang S DeGuzman A Bucana CD Fidler IJ. Nuclear factor-kappaB Activity Correlates With Growth, Angiogenesis, and Metastasis of Human Melanoma Cells in Nude Mice. Clin Cancer Res (2000) 6:2573–81. 10873114

[94] Schummer P Kuphal S Vardimon L Bosserhoff AK Kappelmann M. Specific C-Jun Target Genes in Malignant Melanoma. Cancer Biol Ther (2016) 17:486–97. doi: 10.1080/15384047.2016.1156264 PMC4910930 27050748

[95] Han YP Downey S Garner WL. Interleukin-1alpha-Induced Proteolytic Activation of Metalloproteinase-9 by Human Skin. Surgery (2005) 138:932–9. doi: 10.1016/j.surg.2005.05.003 PMC2366888 16291395

[96] Cotignola J Reva B Mitra N Ishill N Chuai S Patel A. Matrix Metalloproteinase-9 (MMP-9) Polymorphisms in Patients With Cutaneous Malignant Melanoma. BMC Med Genet (2007) 8:10. doi: 10.1186/1471-2350-8-10 17346338 PMC1831467

[97] Jung YJ Isaacs JS Lee S Trepel J Neckers L. IL-1beta-Mediated Up-Regulation of HIF-1alpha via an NFkappaB/COX-2 Pathway Identifies HIF-1 as a Critical Link Between Inflammation and Oncogenesis. FASEB J (2003) 17:2115–7. doi: 10.1096/fj.03-0329fje 12958148

[98] Linnskog R Jonsson G Axelsson L Prasad CP Andersson T. Interleukin-6 Drives Melanoma Cell Motility Through P38alpha-MAPK-Dependent Up-Regulation of WNT5A Expression. Mol Oncol (2014) 8:1365–78. doi: 10.1016/j.molonc.2014.05.008 PMC5528610 24954857

[99] Chafe SC McDonald PC Saberi S Nemirovsky O Venkateswaran G Burugu S. Targeting Hypoxia-Induced Carbonic Anhydrase IX Enhances Immune-Checkpoint Blockade Locally and Systemically. Cancer Immunol Res (2019) 7:1064–78. doi: 10.1158/2326-6066.CIR-18-0657 31088846

[100] Peppicelli S Andreucci E Ruzzolini J Bianchini F Nediani C Supuran CT. The Carbonic Anhydrase IX Inhibitor SLC-0111 as Emerging Agent Against the Mesenchymal Stem Cell-Derived Pro-Survival Effects on Melanoma Cells. J Enzyme Inhib Med Chem (2020) 35:1185–93. doi: 10.1080/14756366.2020.1764549 PMC7269050 32396749

[101] Louphrasitthiphol P Ledaki I Chauhan J Falletta P Siddaway R Buffa FM. MITF Controls the TCA Cycle to Modulate the Melanoma Hypoxia Response. Pigment Cell Melanoma Res (2019) 32:792–808. doi: 10.1111/pcmr.12802 31207090 PMC6777998

[102] Javelaud D Alexaki VI Pierrat MJ Hoek KS Dennler S Van Kempen L. GLI2 and M-MITF Transcription Factors Control Exclusive Gene Expression Programs and Inversely Regulate Invasion in Human Melanoma Cells. Pigment Cell Melanoma Res (2011) 24:932–43. doi: 10.1111/j.1755-148X.2011.00893.x 21801332

[103] Boshuizen J Vredevoogd DW Krijgsman O Ligtenberg MA Blankenstein S de Bruijn B. Reversal of Pre-Existing NGFR-Driven Tumor and Immune Therapy Resistance. Nat Commun (2020) 11:3946. doi: 10.1038/s41467-020-17739-8 32770055 PMC7414147

[104] Reinhardt J Landsberg J Schmid-Burgk JL Ramis BB Bald T Glodde N. MAPK Signaling and Inflammation Link Melanoma Phenotype Switching to Induction of CD73 During Immunotherapy. Cancer Res (2017) 77:4697–709. doi: 10.1158/0008-5472.CAN-17-0395 28652246

[105] Liu D Lin JR Robitschek EJ Kasumova GG Heyde A Shi A. Evolution of Delayed Resistance to Immunotherapy in a Melanoma Responder. Nat Med (2021) 27:985–92. doi: 10.1038/s41591-021-01331-8 PMC8474080 33941922

[106] Arozarena I Sanchez-Laorden B Packer L Hidalgo-Carcedo C Hayward R Viros A. Oncogenic BRAF Induces Melanoma Cell Invasion by Downregulating the cGMP-Specific Phosphodiesterase PDE5A. Cancer Cell (2011) 19:45–57. doi: 10.1016/j.ccr.2010.10.029 21215707

[107] Goodall J Carreira S Denat L Kobi D Davidson I Nuciforo P. The Brn-2 Transcription Factor Links Activated BRAF to Melanoma Proliferation. Mol Cell Biol (2004) 24:2923–31. doi: 10.1128/MCB.24.7.2923-2931.2004 PMC371133 15024080

[108] Bonvin E Falletta P Shaw H Delmas V Goding CR. A Phosphatidylinositol 3-Kinase-Pax3 Axis Regulates Brn-2 Expression in Melanoma. Mol Cell Biol (2012) 32:4674–83. doi: 10.1128/MCB.01067-12 PMC3486186 22988297

[109] Berlin I Denat L Steunou AL Puig I Champeval D Colombo S. Phosphorylation of BRN2 Modulates its Interaction With the Pax3 Promoter to Control Melanocyte Migration and Proliferation. Mol Cell Biol (2012) 32:1237–47. doi: 10.1128/MCB.06257-11 PMC3302439 22290434

[110] Goodall J Martinozzi S Dexter TJ Champeval D Carreira S Larue L. Brn-2 Expression Controls Melanoma Proliferation and Is Directly Regulated by Beta-Catenin. Mol Cell Biol (2004) 24:2915–22. doi: 10.1128/MCB.24.7.2915-2922.2004 PMC371132 15024079

[111] Perotti V Baldassari P Molla A Vegetti C Bersani I Maurichi A. NFATc2 Is an Intrinsic Regulator of Melanoma Dedifferentiation. Oncogene (2016) 35:2862–72. doi: 10.1038/onc.2015.355 26387540

[112] Yasumizu Y Rajabi H Jin C Hata T Pitroda S Long MD. MUC1-C Regulates Lineage Plasticity Driving Progression to Neuroendocrine Prostate Cancer. Nat Commun (2020) 11:338. doi: 10.1038/s41467-019-14219-6 31953400 PMC6969104

[113] Fane ME Chhabra Y Hollingsworth DEJ Simmons JL Spoerri L Oh TG. NFIB Mediates BRN2 Driven Melanoma Cell Migration and Invasion Through Regulation of EZH2 and MITF. EBioMedicine (2017) 16:63–75. doi: 10.1016/j.ebiom.2017.01.013 28119061 PMC5474438

[114] Zingg D Debbache J Pena-Hernandez R Antunes AT Schaefer SM Cheng PF. EZH2-Mediated Primary Cilium Deconstruction Drives Metastatic Melanoma Formation. Cancer Cell (2018) 34:69–84.e14. doi: 10.1016/j.ccell.2018.06.001 30008323

[115] Kong X Kuilman T Shahrabi A Boshuizen J Kemper K Song JY. Cancer Drug Addiction Is Relayed by an ERK2-Dependent Phenotype Switch. Nature (2017) 550:270–4. doi: 10.1038/nature24037 PMC5640985 28976960

[116] Karin M. The Regulation of AP-1 Activity by Mitogen-Activated Protein Kinases. J Biol Chem (1995) 270:16483–6. doi: 10.1074/jbc.270.28.16483 7622446

[117] Shaulian E Karin M. AP-1 in Cell Proliferation and Survival. Oncogene (2001) 20:2390–400. doi: 10.1038/sj.onc.1204383 11402335

[118] Nishita M Itsukushima S Nomachi A Endo M Wang Z Inaba D. Ror2/Frizzled Complex Mediates Wnt5a-Induced AP-1 Activation by Regulating Dishevelled Polymerization. Mol Cell Biol (2010) 30:3610–9. doi: 10.1128/MCB.00177-10 PMC2897551 20457807

[119] Pukrop T Klemm F Hagemann T Gradl D Schulz M Siemes S. Wnt 5a Signaling is Critical for Macrophage-Induced Invasion of Breast Cancer Cell Lines. Proc Natl Acad Sci USA (2006) 103:5454–9. doi: 10.1073/pnas.0509703103 PMC1459376 16569699

[120] Weeraratna AT Jiang Y Hostetter G Rosenblatt K Duray P Bittner M. Wnt5a Signaling Directly Affects Cell Motility and Invasion of Metastatic Melanoma. Cancer Cell (2002) 1:279–88. doi: 10.1016/s1535-6108(02)00045-4 12086864

[121] Zhang B. CD73: A Novel Target for Cancer Immunotherapy. Cancer Res (2010) 70:6407–11. doi: 10.1158/0008-5472.CAN-10-1544 PMC2922475 20682793

[122] Huh HD Kim DH Jeong HS Park HW. Regulation of TEAD Transcription Factors in Cancer Biology. Cells (2019) 8(6):600. doi: 10.3390/cells8060600 PMC6628201 31212916

[123] Koo JH Plouffe SW Meng Z Lee DH Yang D Lim DS. Induction of AP-1 by YAP/TAZ Contributes to Cell Proliferation and Organ Growth. Genes Dev (2020) 34:72–86. doi: 10.1101/gad.331546.119 31831627 PMC6938666

[124] Liu X Li H Rajurkar M Li Q Cotton JL Ou J. Tead and AP1 Coordinate Transcription and Motility. Cell Rep (2016) 14:1169–80. doi: 10.1016/j.celrep.2015.12.104 PMC4749442 26832411

[125] Zanconato F Forcato M Battilana G Azzolin L Quaranta E Bodega B. Genome-Wide Association Between YAP/TAZ/TEAD and AP-1 at Enhancers Drives Oncogenic Growth. Nat Cell Biol (2015) 17:1218–27. doi: 10.1038/ncb3216 PMC6186417 26258633

[126] Xu MZ Chan SW Liu AM Wong KF Fan ST Chen J. AXL Receptor Kinase Is a Mediator of YAP-Dependent Oncogenic Functions in Hepatocellular Carcinoma. Oncogene (2011) 30:1229–40. doi: 10.1038/onc.2010.504 PMC3330262 21076472

[127] Zhang X Yang L Szeto P Abali GK Zhang Y Kulkarni A. The Hippo Pathway Oncoprotein YAP Promotes Melanoma Cell Invasion and Spontaneous Metastasis. Oncogene (2020) 39:5267–81. doi: 10.1038/s41388-020-1362-9 32561850

[128] Kim MH Kim J Hong H Lee SH Lee JK Jung E. Actin Remodeling Confers BRAF Inhibitor Resistance to Melanoma Cells Through YAP/TAZ Activation. EMBO J (2016) 35:462–78. doi: 10.15252/embj.201592081 PMC4772854 26668268

[129] Castro DS Skowronska-Krawczyk D Armant O Donaldson IJ Parras C Hunt C. Proneural bHLH and Brn Proteins Coregulate a Neurogenic Program Through Cooperative Binding to a Conserved DNA Motif. Dev Cell (2006) 11:831–44. doi: 10.1016/j.devcel.2006.10.006 17141158

[130] Fane M Chhabra Y Spoerri L Goding C. Reciprocal Regulation of BRN2 and NOTCH1/2 Signaling Synergistically Drives Melanoma Cell Migration and Invasion. J Invest Dermatol (2021). 10.1016/j.jid.2020.12.039 34958806

[131] Noda S Yashiro M Nshii T Hirakawa K. Hypoxia Upregulates Adhesion Ability to Peritoneum Through a Transforming Growth Factor-Beta-Dependent Mechanism in Diffuse-Type Gastric Cancer Cells. Eur J Cancer (2010) 46:995–1005. doi: 10.1016/j.ejca.2010.01.007 20144860

[132] Copple BL. Hypoxia Stimulates Hepatocyte Epithelial to Mesenchymal Transition by Hypoxia-Inducible Factor and Transforming Growth Factor-Beta-Dependent Mechanisms. Liver Int (2010) 30:669–82. doi: 10.1111/j.1478-3231.2010.02205.x PMC3111074 20158611

[133] Dissanayake SK Olkhanud PB O'Connell MP Carter A French AD Camilli TC. Wnt5A Regulates Expression of Tumor-Associated Antigens in Melanoma via Changes in Signal Transducers and Activators of Transcription 3 Phosphorylation. Cancer Res (2008) 68:10205–14. doi: 10.1158/0008-5472.CAN-08-2149 PMC2605679 19074888

[134] Holtzhausen A Zhao F Evans KS Tsutsui M Orabona C Tyler DS. Melanoma-Derived Wnt5a Promotes Local Dendritic-Cell Expression of IDO and Immunotolerance: Opportunities for Pharmacologic Enhancement of Immunotherapy. Cancer Immunol Res (2015) 3:1082–95. doi: 10.1158/2326-6066.CIR-14-0167 PMC4927300 26041736

[135] Zingg D Debbache J Schaefer SM Tuncer E Frommel SC Cheng P. The Epigenetic Modifier EZH2 Controls Melanoma Growth and Metastasis Through Silencing of Distinct Tumour Suppressors. Nat Commun (2015) 6:6051. doi: 10.1038/ncomms7051 25609585

[136] Perotti V Baldassari P Molla A Nicolini G Bersani I Grazia G. An Actionable Axis Linking NFATc2 to EZH2 Controls the EMT-Like Program of Melanoma Cells. Oncogene (2019) 38:4384–96. doi: 10.1038/s41388-019-0729-2 PMC6756060 30710146

[137] Boshuizen J Pencheva N Krijgsman O Altimari DD Castro PG de Bruijn B. Cooperative Targeting of Melanoma Heterogeneity With an AXL Antibody-Drug Conjugate and BRAF/MEK Inhibitors. Nat Med (2018) 24:203–12. doi: 10.1038/nm.4472 29334371

[138] Boshuizen J Pencheva N Krijgsman O Altimari DD Castro PG de Bruijn B. Cooperative Targeting of Immunotherapy-Resistant Melanoma and Lung Cancer by an AXL-Targeting Antibody-Drug Conjugate and Immune Checkpoint Blockade. Cancer Res (2021) 81:1775–87. doi: 10.1158/0008-5472.CAN-20-0434 33531370

[139] Marin-Bejar O Rogiers A Dewaele M Femel J Karras P Pozniak J. Evolutionary Predictability of Genetic Versus Nongenetic Resistance to Anticancer Drugs in Melanoma. Cancer Cell (2021) 39:1135–49.e1138. doi: 10.1016/j.ccell.2021.05.015 34143978

[140] Feng WW Wilkins O Bang S Ung M Li J An J. CD36-Mediated Metabolic Rewiring of Breast Cancer Cells Promotes Resistance to HER2-Targeted Therapies. Cell Rep (2019) 29:3405–20.e3405. doi: 10.1016/j.celrep.2019.11.008 31825825 PMC6938262

[141] Drury J Rychahou PG He D Jafari N Wang C Lee EY. Inhibition of Fatty Acid Synthase Upregulates Expression of CD36 to Sustain Proliferation of Colorectal Cancer Cells. Front Oncol (2020) 10:1185. doi: 10.3389/fonc.2020.01185 32850342 PMC7411002

[142] Hou Y Wu M Wei J Ren Y Du C Wu H. CD36 is Involved in High Glucose-Induced Epithelial to Mesenchymal Transition in Renal Tubular Epithelial Cells. Biochem Biophys Res Commun (2015) 468:281–6. doi: 10.1016/j.bbrc.2015.10.112 26505798

[143] Pascual G Avgustinova A Mejetta S Martin M Castellanos A Attolini CS. Targeting Metastasis-Initiating Cells Through the Fatty Acid Receptor CD36. Nature (2017) 541:41–5. doi: 10.1038/nature20791 27974793

[144] Shen S Faouzi S Bastide A Martineau S Malka-Mahieu H Fu Y. An Epitranscriptomic Mechanism Underlies Selective mRNA Translation Remodelling in Melanoma Persister Cells. Nat Commun (2019) 10:5713. doi: 10.1038/s41467-019-13360-6 31844050 PMC6915789

[145] Boussemart L Malka-Mahieu H Girault I Allard D Hemmingsson O Tomasic G. Eif4f Is a Nexus of Resistance to Anti-BRAF and Anti-MEK Cancer Therapies. Nature (2014) 513:105–9. doi: 10.1038/nature13572 25079330

[146] Huang F Goncalves C Bartish M Remy-Sarrazin J Issa ME Cordeiro B. Inhibiting the MNK1/2-Eif4e Axis Impairs Melanoma Phenotype Switching and Potentiates Antitumor Immune Responses. J Clin Invest (2021) 131(8):e140752. doi: 10.1172/JCI140752 PMC8262472 33690225

[147] Harding HP Zhang Y Zeng H Novoa I Lu PD Calfon M. An Integrated Stress Response Regulates Amino Acid Metabolism and Resistance to Oxidative Stress. Mol Cell (2003) 11:619–33. doi: 10.1016/s1097-2765(03)00105-9 12667446

[148] Garcia-Jimenez C Goding CR. Starvation and Pseudo-Starvation as Drivers of Cancer Metastasis Through Translation Reprogramming. Cell Metab (2019) 29:254–67. doi: 10.1016/j.cmet.2018.11.018 PMC6365217 30581118

[149] Robichaud N del Rincon SV Huor B Alain T Petruccelli LA Hearnden J. Phosphorylation of Eif4e Promotes EMT and Metastasis via Translational Control of SNAIL and MMP-3. Oncogene (2015) 34:2032–42. doi: 10.1038/onc.2014.146 PMC4978545 24909168

[150] Gkogkas CG Khoutorsky A Cao R Jafarnejad SM Prager-Khoutorsky M Giannakas N. Pharmacogenetic Inhibition of Eif4e-Dependent Mmp9 mRNA Translation Reverses Fragile X Syndrome-Like Phenotypes. Cell Rep (2014) 9:1742–55. doi: 10.1016/j.celrep.2014.10.064 PMC4294557 25466251

[151] Shoshan E Braeuer RR Kamiya T Mobley AK Huang L Vasquez ME. NFAT1 Directly Regulates IL8 and MMP3 to Promote Melanoma Tumor Growth and Metastasis. Cancer Res (2016) 76:3145–55. doi: 10.1158/0008-5472.CAN-15-2511 PMC4891299 27013197

[152] Fang R Zhang G Guo Q Ning F Wang H Cai S. Nodal Promotes Aggressive Phenotype via Snail-Mediated Epithelial-Mesenchymal Transition in Murine Melanoma. Cancer Lett (2013) 333:66–75. doi: 10.1016/j.canlet.2013.01.014 23348697

[153] Fu S Zhang N Yopp AC Chen D Mao M Chen D. TGF-Beta Induces Foxp3 + T-Regulatory Cells From CD4 + CD25 - Precursors. Am J Transplant (2004) 4:1614–27. doi: 10.1111/j.1600-6143.2004.00566.x 15367216

[154] Valastyan S Weinberg RA. Tumor Metastasis: Molecular Insights and Evolving Paradigms. Cell (2011) 147:275–92. doi: 10.1016/j.cell.2011.09.024 PMC3261217 22000009

[155] Lugassy C Wadehra M Li X Corselli M Akhavan D Binder SW. Pilot Study on "Pericytic Mimicry" and Potential Embryonic/Stem Cell Properties of Angiotropic Melanoma Cells Interacting With the Abluminal Vascular Surface. Cancer Microenviron (2013) 6:19–29. doi: 10.1007/s12307-012-0128-5 23275074 PMC3601217

[156] Bertrand F Rochotte J Colacios C Montfort A Tilkin-Mariame AF Touriol C. Blocking Tumor Necrosis Factor Alpha Enhances CD8 T-Cell-Dependent Immunity in Experimental Melanoma. Cancer Res (2015) 75:2619–28. doi: 10.1158/0008-5472.CAN-14-2524 25977337

[157] Knol AC Nguyen JM Quereux G Brocard A Khammari A Dreno B. Prognostic Value of Tumor-Infiltrating Foxp3+ T-Cell Subpopulations in Metastatic Melanoma. Exp Dermatol (2011) 20:430–4. doi: 10.1111/j.1600-0625.2011.01260.x 21410773

[158] Santinon F Batignes M Mebrek ML Biton J Clavel G Herve R. Involvement of Tumor Necrosis Factor Receptor Type II in FoxP3 Stability and as a Marker of Treg Cells Specifically Expanded by Anti-Tumor Necrosis Factor Treatments in Rheumatoid Arthritis. Arthritis Rheumatol (2020) 72:576–87. doi: 10.1002/art.41134 31609517

[159] Leveen P Larsson J Ehinger M Cilio CM Sundler M Sjostrand LJ. Induced Disruption of the Transforming Growth Factor Beta Type II Receptor Gene in Mice Causes a Lethal Inflammatory Disorder That is Transplantable. Blood (2002) 100:560–8. doi: 10.1182/blood.v100.2.560 12091349

[160] Rodeck U Bossler A Graeven U Fox FE Nowell PC Knabbe C.Transforming Growth Factor Beta Production and Responsiveness in Normal Human Melanocytes and Melanoma Cells. Cancer Res (1994) 54(2):575–81. 8275496

[161] Granados-Principal S Liu Y Guevara ML Blanco E Choi DS Qian W. Inhibition of iNOS as a Novel Effective Targeted Therapy Against Triple-Negative Breast Cancer. Breast Cancer Res (2015) 17:25. doi: 10.1186/s13058-015-0527-x 25849745 PMC4384389

[162] Pierce CJ Simmons JL Broit N Karunarathne D Ng MF Boyle GM. BRN2 Expression Increases Anoikis Resistance in Melanoma. Oncogenesis (2020) 9:64. doi: 10.1038/s41389-020-00247-1 32632141 PMC7338542

[163] Mauviel A Chung KY Agarwal A Tamai K Uitto J. Cell-Specific Induction of Distinct Oncogenes of the Jun Family Is Responsible for Differential Regulation of Collagenase Gene Expression by Transforming Growth Factor-Beta in Fibroblasts and Keratinocytes. J Biol Chem (1996) 271:10917–23. doi: 10.1074/jbc.271.18.10917 8631909

[164] Wan X Zhu Y Zhang L Hou W. Gefitinib Inhibits Malignant Melanoma Cells Through the VEGF/AKT Signaling Pathway. Mol Med Rep (2018) 17:7351–5. doi: 10.3892/mmr.2018.8728 29568946

[165] Boyle GM Pedley J Martyn AC Banducci KJ Strutton GM Brown DA. Macrophage Inhibitory Cytokine-1 Is Overexpressed in Malignant Melanoma and is Associated With Tumorigenicity. J Invest Dermatol (2009) 129:383–91. doi: 10.1038/jid.2008.270 18754039

[166] Viel S Marcais A Guimaraes FS Loftus R Rabilloud J Grau M. TGF-Beta Inhibits the Activation and Functions of NK Cells by Repressing the mTOR Pathway. Sci Signal (2016) 9:ra19. doi: 10.1126/scisignal.aad1884 26884601

[167] Thomas DA Massague J. TGF-Beta Directly Targets Cytotoxic T Cell Functions During Tumor Evasion of Immune Surveillance. Cancer Cell (2005) 8:369–80. doi: 10.1016/j.ccr.2005.10.012 16286245

[168] Evavold CL Ruan J Tan Y Xia S Wu H Kagan JC. The Pore-Forming Protein Gasdermin D Regulates Interleukin-1 Secretion From Living Macrophages. Immunity (2018) 48:35–44.e36. doi: 10.1016/j.immuni.2017.11.013 29195811 PMC5773350

[169] Yang Y Wang H Kouadir M Song H Shi F. Recent Advances in the Mechanisms of NLRP3 Inflammasome Activation and its Inhibitors. Cell Death Dis (2019) 10:128. doi: 10.1038/s41419-019-1413-8 30755589 PMC6372664

[170] Yurkovetsky ZR Kirkwood JM Edington HD Marrangoni AM Velikokhatnaya L Winans MT. Multiplex Analysis of Serum Cytokines in Melanoma Patients Treated With Interferon-Alpha2b. Clin Cancer Res (2007) 13:2422–8. doi: 10.1158/1078-0432.CCR-06-1805 17438101

[171] Kholmanskikh O van Baren N Brasseur F Ottaviani S Vanacker J Arts N. Interleukins 1alpha and 1beta Secreted by Some Melanoma Cell Lines Strongly Reduce Expression of MITF-M and Melanocyte Differentiation Antigens. Int J Cancer (2010) 127:1625–36. doi: 10.1002/ijc.25182 20099279

[172] Gubernatorova EO Gorshkova EA Namakanova OA Zvartsev RV Hidalgo J Drutskaya MS. Non-Redundant Functions of IL-6 Produced by Macrophages and Dendritic Cells in Allergic Airway Inflammation. Front Immunol (2018) 9:2718. doi: 10.3389/fimmu.2018.02718 30534125 PMC6276801

[173] Hooper WC Phillips DJ Renshaw MA Evatt BL Benson JM. The Up-Regulation of IL-6 and IL-8 in Human Endothelial Cells by Activated Protein C. J Immunol (1998) 161:2567–73. 9725257

[174] Korn T Mitsdoerffer M Croxford AL Awasthi A Dardalhon VA Galileos G. IL-6 Controls Th17 Immunity In Vivo by Inhibiting the Conversion of Conventional T Cells Into Foxp3+ Regulatory T Cells. Proc Natl Acad Sci USA (2008) 105:18460–5. doi: 10.1073/pnas.0809850105 PMC2587589 19015529

[175] Crotty S. T Follicular Helper Cell Differentiation, Function, and Roles in Disease. Immunity (2014) 41:529–42. doi: 10.1016/j.immuni.2014.10.004 PMC4223692 25367570

[176] Takeda K Kaisho T Yoshida N Takeda J Kishimoto T Akira S. Stat3 Activation is Responsible for IL-6-Dependent T Cell Proliferation Through Preventing Apoptosis: Generation and Characterization of T Cell-Specific Stat3-Deficient Mice. J Immunol (1998) 161:4652–60. doi: 10.1038/s41591-018-0217-1 9794394

[177] Ludwig H Nachbaur DM Fritz E Krainer M Huber H. Interleukin-6 Is a Prognostic Factor in Multiple Myeloma. Blood (1991) 77:2794–5. doi: 10.1182/blood.V77.12.2794.2794 2043775

[178] Lee LJ Papadopoli D Jewer M Del Rincon S Topisirovic I Lawrence MG. Cancer Plasticity: The Role of mRNA Translation. Trends Cancer (2021) 7:134–45. doi: 10.1016/j.trecan.2020.09.005 PMC8023421 33067172

[179] Sonenberg N Hinnebusch AG. Regulation of Translation Initiation in Eukaryotes: Mechanisms and Biological Targets. Cell (2009) 136:731–45. doi: 10.1016/j.cell.2009.01.042 PMC3610329 19239892

[180] Furic L Rong L Larsson O Koumakpayi IH Yoshida K Brueschke A. Eif4e Phosphorylation Promotes Tumorigenesis and Is Associated With Prostate Cancer Progression. Proc Natl Acad Sci USA (2010) 107:14134–9. doi: 10.1073/pnas.1005320107 PMC2922605 20679199

[181] Pyronnet S Imataka H Gingras AC Fukunaga R Hunter T Sonenberg N. Human Eukaryotic Translation Initiation Factor 4G (Eif4g) Recruits Mnk1 to Phosphorylate Eif4e. EMBO J (1999) 18:270–9. doi: 10.1093/emboj/18.1.270 PMC1171121 9878069

[182] Yang W Khoury E Guo Q Prabhu SA Emond A Huang F. MNK1 Signaling Induces an ANGPTL4-Mediated Gene Signature to Drive Melanoma Progression. Oncogene (2020) 39:3650–65. doi: 10.1038/s41388-020-1240-5 32132651

[183] Zhou J Jin B Jin Y Liu Y Pan J. The Antihelminthic Drug Niclosamide Effectively Inhibits the Malignant Phenotypes of Uveal Melanoma In Vitro and In Vivo. Theranostics (2017) 7:1447–62. doi: 10.7150/thno.17451 PMC5436505 28529629

[184] Guo Q Li VZ Nichol JN Huang F Yang W Preston SEJ. MNK1/NODAL Signaling Promotes Invasive Progression of Breast Ductal Carcinoma In Situ. Cancer Res (2019) 79:1646–57. doi: 10.1158/0008-5472.CAN-18-1602 PMC6513674 30659022

[185] Smith MP Brunton H Rowling EJ Ferguson J Arozarena I Miskolczi Z. Inhibiting Drivers of Non-Mutational Drug Tolerance Is a Salvage Strategy for Targeted Melanoma Therapy. Cancer Cell (2016) 29:270–84. doi: 10.1016/j.ccell.2016.02.003 PMC4796027 26977879

[186] Smith MP Ferguson J Arozarena I Hayward R Marais R Chapman A. Effect of SMURF2 Targeting on Susceptibility to MEK Inhibitors in Melanoma. J Natl Cancer Inst (2013) 105:33–46. doi: 10.1093/jnci/djs471 23250956 PMC3536641

[187] Haq R Shoag J Andreu-Perez P Yokoyama S Edelman H Rowe GC. Oncogenic BRAF Regulates Oxidative Metabolism via PGC1alpha and MITF. Cancer Cell (2013) 23:302–15. doi: 10.1016/j.ccr.2013.02.003 PMC3635826 23477830

[188] Hugo W Shi H Sun L Piva M Song C Kong X. Non-Genomic and Immune Evolution of Melanoma Acquiring MAPKi Resistance. Cell (2015) 162:1271–85. doi: 10.1016/j.cell.2015.07.061 PMC4821508 26359985

[189] Konieczkowski DJ Johannessen CM Abudayyeh O Kim JW Cooper ZA Piris A. A Melanoma Cell State Distinction Influences Sensitivity to MAPK Pathway Inhibitors. Cancer Discov (2014) 4:816–27. doi: 10.1158/2159-8290.CD-13-0424 PMC4154497 24771846

[190] Kozar I Margue C Rothengatter S Haan C Kreis S. Many Ways to Resistance: How Melanoma Cells Evade Targeted Therapies. Biochim Biophys Acta Rev Cancer (2019) 1871:313–22. doi: 10.1016/j.bbcan.2019.02.002 30776401

[191] Ouzounova M Lee E Piranlioglu R Andaloussi A Kolhe R Demirci MF. Monocytic and Granulocytic Myeloid Derived Suppressor Cells Differentially Regulate Spatiotemporal Tumour Plasticity During Metastatic Cascade. Nat Commun (2017) 8:14979. doi: 10.1038/ncomms14979 28382931 PMC5384228

[192] Douglass SM Fane ME Sanseviero E Ecker BL Kugel CH 3rd Behera R. Myeloid-Derived Suppressor Cells Are a Major Source of Wnt5A in the Melanoma Microenvironment and Depend on Wnt5A for Full Suppressive Activity. Cancer Res (2021) 81:658–70. doi: 10.1158/0008-5472.CAN-20-1238 PMC8330365 33262126

[193] Kumar V Patel S Tcyganov E Gabrilovich DI. The Nature of Myeloid-Derived Suppressor Cells in the Tumor Microenvironment. Trends Immunol (2016) 37:208–20. doi: 10.1016/j.it.2016.01.004 PMC4775398 26858199

[194] Soudja SM Wehbe M Mas A Chasson L de Tenbossche CP Huijbers I. Tumor-Initiated Inflammation Overrides Protective Adaptive Immunity in an Induced Melanoma Model in Mice. Cancer Res (2010) 70:3515–25. doi: 10.1158/0008-5472.CAN-09-4354 20406967

[195] Wehbe M Soudja SM Mas A Chasson L Guinamard R de Tenbossche CP. Epithelial-Mesenchymal-Transition-Like and TGFbeta Pathways Associated With Autochthonous Inflammatory Melanoma Development in Mice. PloS One (2012) 7:e49419. doi: 10.1371/journal.pone.0049419 23173060 PMC3500287

[196] Furuta J Inozume T Harada K Shimada S. CD271 on Melanoma Cell is an IFN-Gamma-Inducible Immunosuppressive Factor That Mediates Downregulation of Melanoma Antigens. J Invest Dermatol (2014) 134:1369–77. doi: 10.1038/jid.2013.490 24226422

[197] Smith MP Wellbrock C. Molecular Pathways: Maintaining MAPK Inhibitor Sensitivity by Targeting Nonmutational Tolerance. Clin Cancer Res (2016) 22:5966–70. doi: 10.1158/1078-0432.CCR-16-0954 PMC5300098 27797970

[198] Yang C Tian C Hoffman TE Jacobsen NK Spencer SL. Melanoma Subpopulations That Rapidly Escape MAPK Pathway Inhibition Incur DNA Damage and Rely on Stress Signalling. Nat Commun (2021) 12:1747. doi: 10.1038/s41467-021-21549-x 33741929 PMC7979728

[199] Guo Q Huang F Goncalves C Del Rincon SV Miller WH Jr. Translation of Cancer Immunotherapy From the Bench to the Bedside. Adv Cancer Res (2019) 143:1–62. doi: 10.1016/bs.acr.2019.03.001 31202357

[200] Sharma P Hu-Lieskovan S Wargo JA Ribas A. Primary, Adaptive, and Acquired Resistance to Cancer Immunotherapy. Cell (2017) 168:707–23. doi: 10.1016/j.cell.2017.01.017 PMC5391692 28187290

[201] Bai R Chen N Li L Du N Bai L Lv Z. Mechanisms of Cancer Resistance to Immunotherapy. Front Oncol (2020) 10:1290. doi: 10.3389/fonc.2020.01290 32850400 PMC7425302

[202] Hugo W Shi H Sun L Piva M Song C Kong X. Genomic and Transcriptomic Features of Response to Anti-PD-1 Therapy in Metastatic Melanoma. Cell (2016) 165:35–44. doi: 10.1016/j.cell.2016.02.065 26997480 PMC4808437

[203] Cerezo M Guemiri R Druillennec S Girault I Malka-Mahieu H Shen S. Translational Control of Tumor Immune Escape via the Eif4f-STAT1-PD-L1 Axis in Melanoma. Nat Med (2018) 24:1877–86. doi: 10.1038/s41591-018-0217-1 30374200

[204] Xu Y Poggio M Jin HY Shi Z Forester CM Wang Y. Translation Control of the Immune Checkpoint in Cancer and Its Therapeutic Targeting. Nat Med (2019) 25:301–11. doi: 10.1038/s41591-018-0321-2 PMC6613562 30643286

[205] Shain AH Garrido M Botton T Talevich E Yeh I Sanborn JZ. Exome Sequencing of Desmoplastic Melanoma Identifies Recurrent NFKBIE Promoter Mutations and Diverse Activating Mutations in the MAPK Pathway. Nat Genet (2015) 47:1194–9. doi: 10.1038/ng.3382 PMC4589486 26343386

[206] Eroglu Z Zaretsky JM Hu-Lieskovan S Kim DW Algazi A Johnson DB. High Response Rate to PD-1 Blockade in Desmoplastic Melanomas. Nature (2018) 553:347–50. doi: 10.1038/nature25187 PMC5773412 29320474

[207] Ansieau S Collin G Hill L. EMT or EMT-Promoting Transcription Factors, Where to Focus the Light? Front Oncol (2014) 4:353. doi: 10.3389/fonc.2014.00353 25566496 PMC4267187

[208] Yang J Manson DK Marr BP Carvajal RD. Treatment of Uveal Melanoma: Where Are We Now? Ther Adv Med Oncol (2018) 10:1758834018757175. doi: 10.1177/1758834018757175 29497459 PMC5824910

[209] Boru G Cebulla CM Sample KM Massengill JB Davidorf FH Abdel-Rahman MH. Heterogeneity in Mitogen-Activated Protein Kinase (MAPK) Pathway Activation in Uveal Melanoma With Somatic GNAQ and GNA11 Mutations. Invest Ophthalmol Vis Sci (2019) 60:2474–80. doi: 10.1167/iovs.18-26452 PMC6557618 31173078

[210] Cruz F 3rd Rubin BP Wilson D Town A Schroeder A Haley A. Absence of BRAF and NRAS Mutations in Uveal Melanoma. Cancer Res (2003) 63:5761–6. 14522897

[211] Triozzi PL Singh AD. Adjuvant Therapy of Uveal Melanoma: Current Status. Ocul Oncol Pathol (2014) 1:54–62. doi: 10.1159/000367715 27175362 PMC4864524

[212] Jager MJ Shields CL Cebulla CM Abdel-Rahman MH Grossniklaus HE Stern MH. Uveal Melanoma. Nat Rev Dis Primers (2020) 6:24. doi: 10.1038/s41572-020-0158-0 32273508

[213] Onken MD Ehlers JP Worley LA Makita J Yokota Y Harbour JW. Functional Gene Expression Analysis Uncovers Phenotypic Switch in Aggressive Uveal Melanomas. Cancer Res (2006) 66:4602–9. doi: 10.1158/0008-5472.CAN-05-4196 PMC5407689 16651410

[214] Asnaghi L Gezgin G Tripathy A Handa JT Merbs SL van der Velden PA. EMT-Associated Factors Promote Invasive Properties of Uveal Melanoma Cells. Mol Vis (2015) 21:919–29. PMC4548792 26321866

[215] Asnaghi L Ebrahimi KB Schreck KC Bar EE Coonfield ML Bell WR. Notch Signaling Promotes Growth and Invasion in Uveal Melanoma. Clin Cancer Res (2012) 18:654–65. doi: 10.1158/1078-0432.CCR-11-1406 PMC4648284 22228632

[216] Mallikarjuna K Pushparaj V Biswas J Krishnakumar S. Expression of Insulin-Like Growth Factor Receptor (IGF-1R), C-Fos, and C-Jun in Uveal Melanoma: An Immunohistochemical Study. Curr Eye Res (2006) 31:875–83. doi: 10.1080/02713680600878790 17050279

[217] Dong L You S Zhang Q Osuka S Devi NS Kaluz S. Arylsulfonamide 64b Inhibits Hypoxia/HIF-Induced Expression of C-Met and CXCR4 and Reduces Primary Tumor Growth and Metastasis of Uveal Melanoma. Clin Cancer Res (2019) 25:2206–18. doi: 10.1158/1078-0432.CCR-18-1368 PMC6445693 30563937

[218] Bustamante P Piquet L Landreville S Burnier JV. Uveal Melanoma Pathobiology: Metastasis to the Liver. Semin Cancer Biol (2021) 71:65–85. doi: 10.1016/j.semcancer.2020.05.003 32450140

[219] Li Y Shi J Yang J Ge S Zhang J Jia R. Uveal Melanoma: Progress in Molecular Biology and Therapeutics. Ther Adv Med Oncol (2020) 12:1758835920965852. doi: 10.1177/1758835920965852 33149769 PMC7586035

[220] Kim YJ Lee SC Kim SE Kim SH Kim SK Lee CS. YAP Activity is Not Associated With Survival of Uveal Melanoma Patients and Cell Lines. Sci Rep (2020) 10:6209. doi: 10.1038/s41598-020-63391-z 32277165 PMC7148330

[221] Li H Li Q Dang K Ma S Cotton JL Yang S. YAP/TAZ Activation Drives Uveal Melanoma Initiation and Progression. Cell Rep (2019) 29:3200–11.e3204. doi: 10.1016/j.celrep.2019.03.021 31801083 PMC7871510

[222] Doherty RE Sisley K Hammond DW Rennie IG Cross NA. Phenotypic Plasticity in Uveal Melanoma Is Not Restricted to a Tumor Subpopulation and Is Unrelated to Cancer Stem Cell Characteristics. Invest Ophthalmol Vis Sci (2017) 58:5387–95. doi: 10.1167/iovs.17-22272 29049740

[223] Djirackor L Kalirai H Coupland SE Petrovski G. CD166high Uveal Melanoma Cells Represent a Subpopulation With Enhanced Migratory Capacity. Invest Ophthalmol Vis Sci (2019) 60:2696–704. doi: 10.1167/iovs.18-26431 31242292

[224] Valyi-Nagy K Kormos B Ali M Shukla D Valyi-Nagy T. Stem Cell Marker CD271 is Expressed by Vasculogenic Mimicry-Forming Uveal Melanoma Cells in Three-Dimensional Cultures. Mol Vis (2012) 18:588–92. PMC3298423 22419851

[225] Saez-Ayala M Montenegro MF Sanchez-Del-Campo L Fernandez-Perez MP Chazarra S Freter R. Directed Phenotype Switching as an Effective Antimelanoma Strategy. Cancer Cell (2013) 24:105–19. doi: 10.1016/j.ccr.2013.05.009 23792190

[226] Chua V Mattei J Han A Johnston L LiPira K Selig SM. The Latest on Uveal Melanoma Research and Clinical Trials: Updates From the Cure Ocular Melanoma (CURE OM) Science Meeting (2019). Clin Cancer Res (2021) 27:28–33. doi: 10.1158/1078-0432.CCR-20-2536 33060121 PMC8213456

[227] Paradis JS Acosta M Saddawi-Konefka R Kishore A Gomes F Arang N. Synthetic Lethal Screens Reveal Cotargeting FAK and MEK as a Multimodal Precision Therapy for GNAQ-Driven Uveal Melanoma. Clin Cancer Res (2021) 27:3190–200. doi: 10.1158/1078-0432.CCR-20-3363 PMC8895627 33568347

[228] Neelature S Sriramareddy Smalley KSM. MEK-Ing the Most of It: Strategies to Co-Target Galphaq and MAPK in Uveal Melanoma. Clin Cancer Res (2021) 27:1217–9. doi: 10.1158/1078-0432.CCR-20-4530 PMC7925419 33355300

[229] Yu FX Luo J Mo JS Liu G Kim YC Meng Z. Mutant Gq/11 Promote Uveal Melanoma Tumorigenesis by Activating YAP. Cancer Cell (2014) 25:822–30. doi: 10.1016/j.ccr.2014.04.017 PMC4075337 24882516

[230] Damato BE Dukes J Goodall H Carvajal RD. Tebentafusp: T Cell Redirection for the Treatment of Metastatic Uveal Melanoma. Cancers (Basel) (2019) 11(7):971. doi: 10.3390/cancers11070971 PMC6679206 31336704

